# Targeted in situ cross-linking mass spectrometry and integrative modeling reveal the architectures of three proteins from SARS-CoV-2

**DOI:** 10.1073/pnas.2103554118

**Published:** 2021-08-09

**Authors:** Moriya Slavin, Joanna Zamel, Keren Zohar, Tsiona Eliyahu, Merav Braitbard, Esther Brielle, Leah Baraz, Miri Stolovich-Rain, Ahuva Friedman, Dana G. Wolf, Alexander Rouvinski, Michal Linial, Dina Schneidman-Duhovny, Nir Kalisman

**Affiliations:** ^a^Department of Biological Chemistry, Institute of Life Sciences, The Hebrew University of Jerusalem, Jerusalem 9190401, Israel;; ^b^Hadassah Academic College Jerusalem, Jerusalem 9101001, Israel;; ^c^Department of Microbiology and Molecular Genetics, Institute for Medical Research Israel-Canada, The Kuvin Center for the Study of Infectious and Tropical Diseases, The Hebrew University-Hadassah Medical School, The Hebrew University of Jerusalem, Jerusalem 9190401, Israel;; ^d^Clinical Virology Unit, Hadassah Hebrew University Medical Center, 9190401 Jerusalem, Israel;; ^e^The Rachel and Selim Benin School of Computer Science and Engineering, The Hebrew University of Jerusalem, Jerusalem 9190401, Israel

**Keywords:** structural biology, mass spectrometry, in-cell techniques, integrative modeling

## Abstract

Atomic structures of several proteins from the coronavirus family are still partial or unavailable. A possible reason for this gap is the instability of these proteins outside of the cellular context, thereby prompting the use of in-cell approaches. In situ cross-linking and mass spectrometry (in situ CLMS) can provide information on the structures of such proteins as they occur in the intact cell. Here, we applied targeted in situ CLMS to structurally probe Nsp1, Nsp2, and nucleocapsid (N) proteins from severe acute respiratory syndrome coronavirus 2 (SARS-CoV-2) and obtained cross-link sets with an average density of one cross-link per 20 residues. We then employed integrative modeling that computationally combined the cross-linking data with domain structures to determine full-length atomic models. For the Nsp2, the cross-links report on a complex topology with long-range interactions. Integrative modeling with structural prediction of individual domains by the AlphaFold2 system allowed us to generate a single consistent all-atom model of the full-length Nsp2. The model reveals three putative metal binding sites and suggests a role for Nsp2 in zinc regulation within the replication–transcription complex. For the N protein, we identified multiple intra- and interdomain cross-links. Our integrative model of the N dimer demonstrates that it can accommodate three single RNA strands simultaneously, both stereochemically and electrostatically. For the Nsp1, cross-links with the 40S ribosome were highly consistent with recent cryogenic electron microscopy structures. These results highlight the importance of cellular context for the structural probing of recalcitrant proteins and demonstrate the effectiveness of targeted in situ CLMS and integrative modeling.

## Significance

We present a generic methodology that extracts structural data from living, intact cells for any protein of interest. Application of this methodology to different viral proteins resulted in significant cross-link sets that revealed the connectivity within their structures. Importantly, we show that these cross-link sets are detailed enough to enable the integrative modeling of the full-length protein sequence. Consequently, we report the global structural organization of Nsp2 and the dimer of the nucleocapsid protein. We foresee that similar applications will be highly useful to study other recalcitrant proteins on which the mainstream structural approaches currently fail.

The genome of severe acute respiratory syndrome coronavirus 2 (SARS-CoV-2) encodes 29 major proteins: 16 nonstructural proteins (Nsp1 to Nsp16), 4 structural proteins (S, E, M, and N), 9 major open reading frames, and several additional noncanonical gene products (1). Despite significant progress in viral protein structure determination (2), there are still gaps in the structural knowledge of several proteins from the coronavirus family (3). For example, the nucleocapsid (N) protein is known to form multisubunit assemblies that were not yet resolved structurally. A possible reason for these difficulties may be the instability of certain viral proteins in the in vitro state. In such cases, purification procedures that are an integral part of mainstream structural approaches (X-ray crystallography, NMR, and cryogenic electron microscopy [cryo-EM]) may cause the purified proteins to disassemble, denature, or aggregate. To avoid such artifacts, in situ techniques for structural studies are required.

In situ cross-linking and mass spectrometry (in situ CLMS) allows probing protein structure inside intact cells (4, 5). In this approach, cells are incubated with a membrane-permeable cross-linking reagent, which reacts with the cellular proteins in their native environment. Following the chemical cross-linking, the cells are lysed and their protein content is analyzed by mass spectrometry (MS). Computational search can then identify from the MS data the pairs of residues that were covalently linked. Because a link between two residues reports on their structural proximity, the list of identified links is a rich resource for modeling protein structures and interactions (6–10). In situ CLMS has progressed significantly in recent years, with applications on isolated organelles (11–15), bacteria (16, 17), human cells (18–20), and heart tissue (21).

An inherent difficulty of in situ CLMS is the high complexity of the initial cell lysates and subsequent tryptic digests. Two general strategies have been employed to reduce the complexity prior to the MS analysis. One strategy enriches the cross-linked peptides out of the total tryptic digest by either tagging the cross-linker itself (21) or by extensive chromatography (16, 17). The other strategy aims to purify a specific protein of interest out of the cell lysate prior to digestion. Wang et al. (18, 19) effectively used the second strategy to study the human proteasome by expressing several of its subunits with a biotin tag. We propose the term “targeted in situ CLMS” to describe the latter approach, which allows the user to focus the MS resources on a small set of predetermined proteins (targets).

In this work we used targeted in situ CLMS and integrative modeling (22, 23) to probe the structures of three SARS-CoV-2 proteins: Nsp1, Nsp2, and the N protein. Our motivation for choosing these proteins is the incomplete knowledge on their structures and functions. For each protein we identified considerable cross-link sets of in situ origin. Computational integration of the cross-links with additional structural information allowed us to build almost complete models for Nsp2 and the N protein.

## Results

### Targeted In Situ CLMS to Study Viral Proteins.

**Fig. 1.**
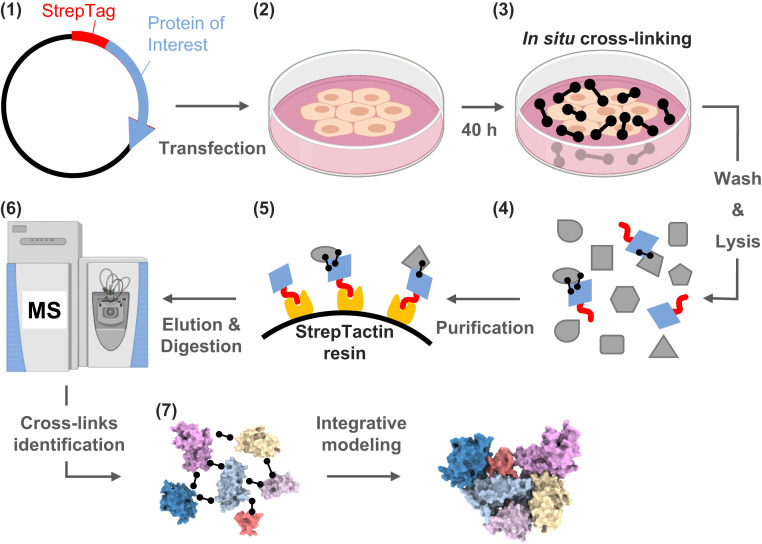
The targeted in situ CLMS workflow for a specific protein of interest. (1) Cloning of a plasmid for constitutive expression of the viral protein with a Strep tag fused at one of the termini. (2) Transfection of human cells in culture with the plasmid, followed by 40 h of expression. (3) The cells are cross-linked in situ by a membrane-permeable reagent (DSS or formaldehyde, black barbells). (4) Washing out the cross-linker before lysis ensures that all the cross-links are of in situ origin. (5) The protein of interest is purified from the lysate by StrepTactin resin. Cross-linked interactors may copurify. (6) MS analyses of the purified proteins reveal the protein composition and identify cross-links. (7) Integrative modeling generates assemblies using domain structural models and cross-links.

We employed a targeted strategy for in situ CLMS of viral proteins inside intact human cells. To that end, HEK293 cells were transfected with a plasmid of a selected viral protein fused to a Strep tag (Fig. 1). We then cross-linked the intact cells with a membrane-permeable cross-linker, washed away the excess cross-linker, lysed the cells, and purified the viral protein via the Strep tag. The purification step greatly enriches and simplifies the sample for MS and increases the subsequent identification rate of cross-links on the target protein.

We focused on three SARS-CoV-2 proteins for which the structural coverage is available only at the domain level: Nsp1 (180 amino acids), Nsp2 (638 amino acids), and the N protein (419 amino acids). The expression levels of all three proteins peaked 40 h after transfection and did not show signs of in-cell aggregation (*SI Appendix*, Fig. S1). Standard proteomics analyses at peak expression (repeated experiments) detected that the N protein was the most abundant in the cell, Nsp2 was detected among the 15 to 30 most abundant proteins, and Nsp1 was among the 250 to 400 most abundant proteins. The cell morphologies appeared normal, but the adherence of the Nsp1-expressing cells to the plate was considerably weaker. These observations are in accordance with the known toxic role of Nsp1, which is mediated through a global translation inhibition (24). Following the Strep purifications, proteomics analyses of the elutions detected the tagged proteins to be the most abundant by a large margin for all three proteins and for both the DSS (disuccinimidyl suberate) and formaldehyde cross-linking reagents (Dataset S1). The Strep tag performance was also satisfactory in two other aspects. First, it enabled repeatable purification yields ranging from ∼5 μg (N protein) to 0.3 μg (Nsp1) from a single plate of cells (*SI Appendix*, Fig. S2), which were sufficient for the MS analyses. Second, the small Strep tag did not seem to interfere with the structures of the expressed proteins (*SI Appendix*, Fig. S3). Overall, we conclude that the Strep tag activity is largely unaffected by amine-reactive cross-linking and suggest it as a tag of choice for such pursuits.

While this methodology is similar to the one introduced by Wang et al. (18, 19), the current protocol was modified in three major aspects: 1) The incubation time of the cells with the cross-linker was shortened to 20 min, rather than 60 min in the original protocol, 2) we used a Strep tag rather than a biotin tag, which allowed us to remove biotinylated proteins from the purification, and 3) we established a transient transfection protocol rather than producing a stable cell line. The transient transfection provides the flexibility of expressing proteins that might be toxic to the cells, such in the case of Nsp1 expression.

### Integrative Modeling of Nsp2 Based on In Situ Cross-Links.

The role of Nsp2 in the viral pathogenicity is poorly understood. Nsp2 is dispensable for viral replication of SARS-CoV in cell culture, although its deletion attenuates viral growth (25). In infected cells, Nsp2 translocates to the double-membrane vesicles (DMVs) in which the replication–transcription complexes (RTCs) are anchored (26). It is yet unclear what the function of Nsp2 is in the context of the DMVs. Secondary structure prediction tools (27) predict that nearly all the sequence of Nsp2 is structured. Yet, this structure remains unknown, thereby making Nsp2 an attractive objective for targeted in situ CLMS.

**Fig. 2.**
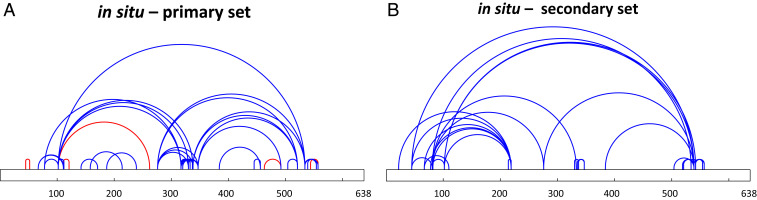
In situ Nsp2 cross-links. (*A*) Cross-links are depicted as arcs (55) on the sequence of Nsp2. Blue and red arcs represent DSS and formaldehyde cross-links, respectively. (*B*) A secondary set of in situ cross-links that were not part of the primary set. The set comprises cross-links in which one of the peptides is short and has poor fragmentation. The secondary set is only used for final selection of models that are otherwise built by restraints from the primary set. The FDR for both sets is 3%.

We targeted Nsp2 for in situ CLMS and analyzed the resulting MS data for proteomics and cross-links. Proteomics analysis revealed 10 proteins that copurify with Nsp2 in significant amounts (*SI Appendix*, Fig. S4*A*). Most of these proteins are part of the Prohibitin complex, which was previously shown to interact with the Nsp2 of SARS-CoV (28). For identification of DSS cross-links, we ran an exhaustive-mode search of the MS data against a sequence database comprising the copurifying proteins and Nsp2. We compiled two nonoverlapping cross-link sets from the search results. The primary set comprised 43 internal cross-links within Nsp2 and 10 cross-links within the Prohibitin complex (Fig. 2*A* and Dataset S2). The secondary set comprised 38 cross-links, which were mostly within Nsp2 (Fig. 2*B* and Dataset S2). The false discovery rate (FDR) for both sets was estimated to be 3% according to a decoy analysis (*Methods* and *SI Appendix*, Figs. S5 and S6). The secondary set only contained cross-links in which one of the peptides was short (four to six residues). Short peptides perform poorly in MS/MS fragmentation, and a common practice in most CLMS studies is to ignore them. However, we found that once a more stringent filtration of the search results is applied these cross-links can be assigned with low FDR values. The primary set is used for structure modeling, while the secondary set is only used for structure validation. In addition to the two sets of DSS cross-links we also identified five cross-links within Nsp2 from formaldehyde cross-linking (Fig. 2*A*) at an FDR of less than 5% according to a decoy analysis with reverse sequences (20).

**Fig. 3.**
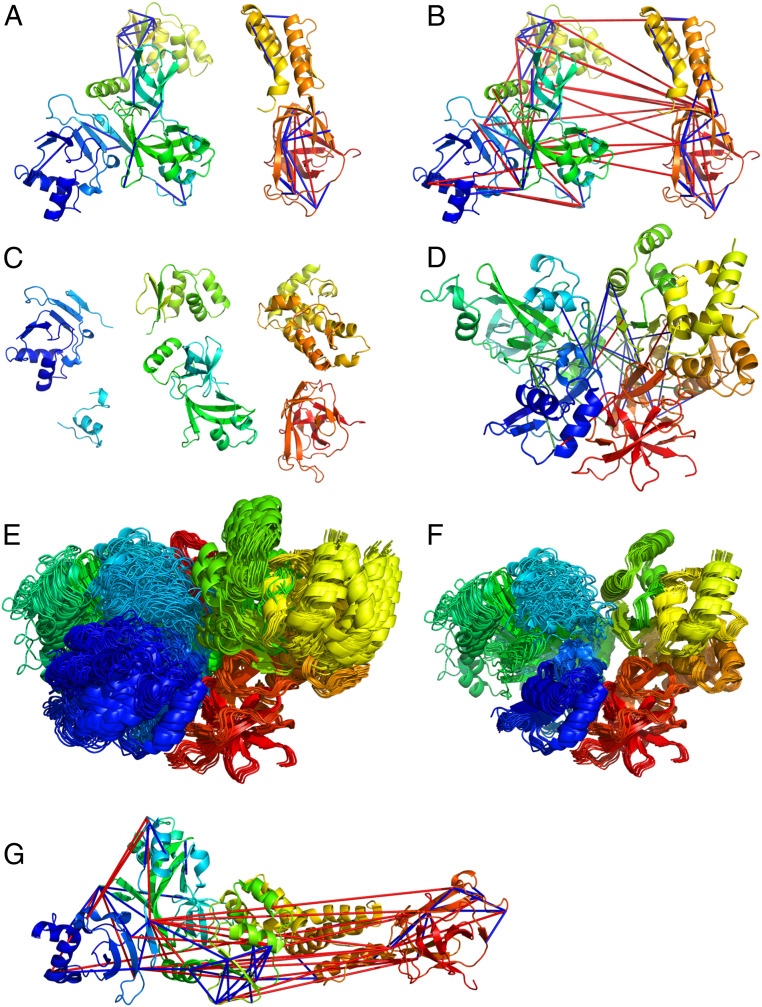
Integrative modeling of Nsp2. The modeling is based on cross-links (satisfied cross-links are in blue, unsatisfied in red). (*A*) Intradomain cross-links mapped onto the AlphaFold2 model. (*B*) All cross-links mapped onto the AlphaFold2 model. (*C*) Six domains that were given as an input to the modeling pipeline. (*D*) Representative result of the integrative modeling with interdomain cross-links (blue, primary set; green, secondary set; red, violated cross-links). (*E*) All models that satisfied the primary set interdomain cross-links. (*F*) Best-scoring models according to the secondary cross-link set. (*G*) Nsp2 cryo-EM structure with in situ cross-links.

There are no homologs with solved structure available for Nsp2. We have therefore referred to the Nsp2 model generated by AlphaFold2 from DeepMind (29). AlphaFold2 has been highly successful in the recent CASP14 round, submitting highly accurate models. The initial AlphaFold2 model violated 17 out of 44 (39%) and 9 out of 29 (31%) cross-links in the primary and secondary sets, respectively. A cross-link is considered violated if the corresponding Cа;–Cа; distance is higher than 25 Å. The violated cross-links were mostly interdomain ones, while almost all the intradomain cross-links were satisfied (Fig. 3 *A* and *B*). To obtain a model consistent with the cross-link set, we divided the AlphaFold2 model into domains (residues 1 to 104, 105 to 132, 133 to 275, 276 to 345, and 512 to 638). One domain that was not covered by the initial AlphaFold2 model (residues 359 to 511) was modeled by homology to partial Nsp2 structure of the infectious bronchitis virus [Protein Data Bank (PDB) ID code 3ld1 (30), sequence identity 13%]. With the availability of the structures for the individual domains, the modeling task is converted into a domain assembly problem. To this end, we applied the CombDock algorithm for multimolecular assembly based on pairwise docking (31–33). The six domains served as an input (Fig. 3*C*) along with the primary set of cross-links and domain connectivity constraints. We have obtained 62 models that satisfied all the primary set interdomain cross-links (Fig. 3 *D* and *E*) with precision of 8 Å. We validated these models with the secondary cross-link set and found nine models that satisfied all but three secondary set cross-links that were in the 25- to 30-Å range. These models converged into a single cluster with a tighter precision of 1 Å (Fig. 3*F*). The precision values for each domain separately were 9.2, 7.8, 6.7, 10.7, 7.8, and 13.2 Å for domains 1 through 6, respectively.

Analysis of the distribution of cysteine and histidine residues in the model identified three putative metal binding sites (*SI Appendix*, Fig. S7). One site is conserved in all coronaviruses, while the two other sites occur only in the SARS subfamily. All three sites are solvent-accessible within the context of the full model and are opposite to the domain that recruits Nsp2 to the RTC. Structural similarity search of the model against the PDB identified several zinc-binding proteins [DALI server (34), Z > 3.0]. Therefore, zinc is the likely ion substrate of Nsp2 as well. Based on these observations we suggest that Nsp2 plays a role in regulation of zinc levels at the RTCs. Zinc is essential for RNA replication and may be depleted at the RTCs, especially when they are enveloped in the DMVs.

During the revision of this article, a cryo-EM structure of Nsp2 was solved (PDB ID code 7msw). The resolution of the density map is 3.76 Å and the structure building also relied on the AlphaFold2 domains. This newer structure violates 25 in situ cross-links (Fig. 3*G*), mainly because its domain architecture is much more open compared to the compact cross-link–based structure (Movie S1). To further study the open and closed conformations of Nsp2, we performed in vitro CLMS under three different cation environments: Zn, Mg, and no cations. The in vitro CLMS protocol first purifies Nsp2 from expressing cells that were not cross-linked and then cross-links the purified protein with the soluble BS3 reagent. For testing the structural effect of the cation environment, the buffers were supplemented with either 50 μM ZnCl2, 50 μM MgCl2, or no cation addition. The three cross-link sets that resulted from the in vitro CLMS experiments were highly similar (*SI Appendix*, Fig. S9), with 75% of the cross-links observed in the intersection of all three sets. The common intersection is consistent (except for one cross-link) with both the open and closed conformations and implies that the individual domains do not require zinc to fold. Interestingly, we observed that cross-links that reported on the closed conformation were much more common in the Zn set. We therefore suggest that Nsp2 exists as a mixture of closed and open states and that zinc binding leads to an elevated population of the closed state. The large number of long-ranged cross-links in the in situ set further suggests that inside the cell the closed state is even more favored.

### Assembly of N Protein Domains Based on In Situ Cross-Links.

**Fig. 4.**
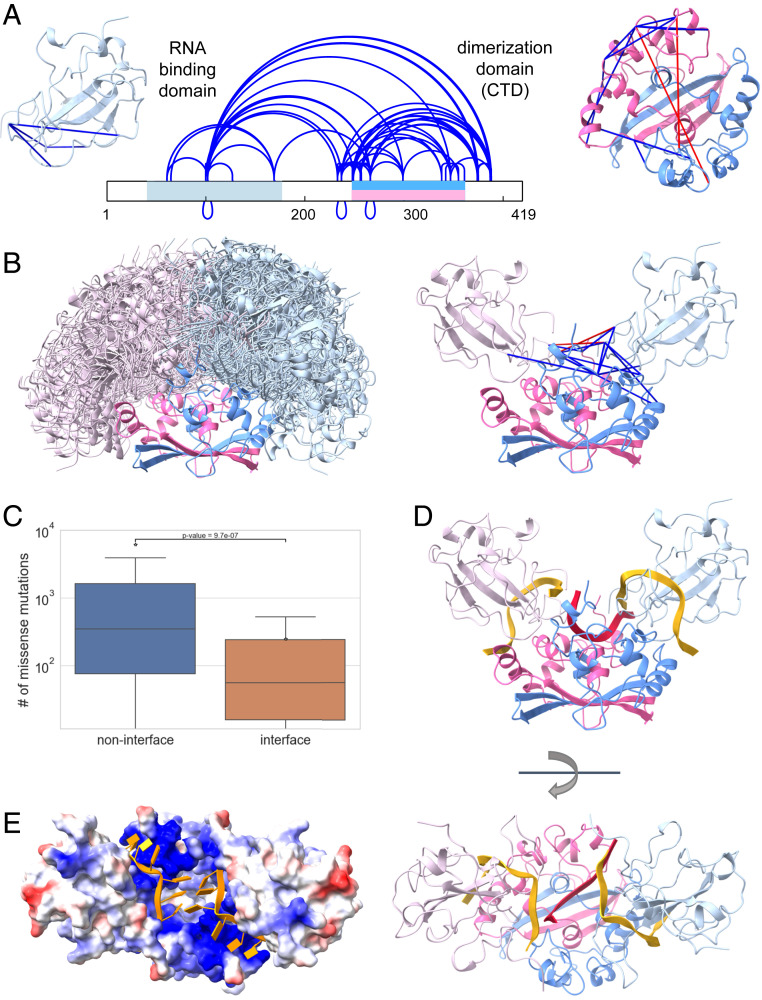
Integrative modeling of the N protein. (*A*) In situ cross-links identified within the N protein. The two structured domains of the protein are marked on the sequence in light blue (RNA binding domain) and pink/blue (dimerization domain). The intradomain cross-links (satisfied cross-links are in blue, unsatisfied in red) are mapped onto the corresponding structures (PDB ID codes 7act^37^ and 6zco^38^ for RNA binding and dimerization domain, respectively). (*B*) Best scoring docking models that satisfy the interdomain cross-links (*Left*) and a representative model (*Right*). (*C*) Number of missense mutations observed in sequenced viral genomes for interface and noninterface residues. (*D*) RNA fragments bound to dimerization domain (red) and RNA binding domains (gold). (*E*) The surface of the N protein colored according to the electrostatics potential with two RNA fragments bound to each of the two RNA binding domains in gold.

The role of the N protein is to pack the viral RNA inside the mature virion. N consists of two domains (RNA binding and dimerization) with known atomic structures for each domain separately (35, 36). These domains are flanked by long linker regions, which are predicted to be largely unstructured (Fig. 4*A*). Recent studies revealed that upon RNA binding N forms higher-order oligomers for tighter packing of the viral RNA inside the capsid (37–40). Yet, the structural details of this oligomerization are still unknown.

Analysis of the proteins that copurify with N suggests that it does not have a direct protein interactor in the human host (*SI Appendix*, Fig. S4*B*). Although no single protein is significantly enriched, the more abundant proteins to copurify all belong to different classes of RNA binders. The most likely explanation is that these proteins bind on the same RNA molecule as N but do not engage in a direct protein–protein interaction. We therefore assume that in HEK239 cells the expressed N protein is at least partially occupied by nonspecific interactions with cytoplasmic RNA.

Identification of in situ DSS cross-links of N (Fig. 4*A* and Dataset S3) followed the same procedure as for Nsp2. We identified 85 unique peptide pairs corresponding to 61 unique cross-linked residue pairs within N and only one cross-link between N and HNRPU, with FDR of 3% (*SI Appendix*, Figs. S5*C* and S6*C*). This interprotein cross-link has a borderline fragmentation score and may in fact be a false positive. The cross-links within N can be divided into three groups. The first group contains 19 intradomain cross-links (4 in the RNA binding domain and 15 in the dimerization domain). Most of these cross-links fit well with the experimental atomic structures (Cа–Cа distance <25 Å), three cross-links are in the 26- to 30-Å range, and two are violated: 266 to 266 (42 Å) and 248 to 249 (30.6 Å) (Fig. 4*A*). These two cross-links most likely belong to higher-order assemblies of the N dimers (37–39, 41). The second group contains the 14 interdomain cross-links, indicating that the RNA binding and dimerization domains directly interact with each other. The third group contains 27 cross-links between the dimerization domain and the linkers, including a few interlinker cross-links.

In order to obtain a model of the full N dimer we performed computational docking (32) of the dimerization domain (in the dimer form) and two RNA binding domains. All three docked components contained short single RNA strands to ensure that the final model is consistent with the paths of bound RNA. The RNA binding domain with bound RNA 10-mer was taken from a recent NMR study [PDB ID code 7act (35)]. The RNA 6-mer on the dimerization domain [PDB ID code 6zco (36)] was initially docked into the basic groove between the monomers and then refined by a molecular dynamics (MD) simulation (*Methods*). The docking was guided by distance restraints derived from the 14 identified interdomain cross-links (33). We obtained a single large cluster of models satisfying all the cross-links within 25 Å, except one (residues 100 to 102) which was within 28 Å (Fig. 4*B*). This cross-link is between two lysines located on a flexible loop, and therefore the distance can vary. The RNA binding domain binds to the dimerization domain at a well-defined region that was largely shared by all the models. This region comprises residues 247 to 261 from one chain and residues 296 to 307 and 343 to 352 from the other chain. To validate the interaction interface between the domains we compared the number of missense mutations in the sequenced SARS-CoV-2 genomes from GISAID in the interface residues vs. noninterface residues (Fig. 4*C*). A residue was defined as an interface residue if it was in contact with the other domain (distance <6 Å from any atom) in at least 50% of the models in the cluster we have obtained. Indeed, there was a significant difference of nearly 10-fold in the average number of mutations between the interface residues and the noninterface ones. This analysis indicates that there is an evolutionary pressure for conservation of this interface.

The integrative model demonstrates that the N dimer can accommodate three RNA single strands simultaneously. Stereochemically, the two RNA binding domains are located far enough from each other to allow a middle RNA single strand to stretch on the basic surface of the dimerization domain without hindrance. We note, however, that a rearrangement of residues 247 to 252 in the dimerization domain is required (compared to current crystal structures) in order to allow the entry and exit of that middle strand. Electrostatically, the closest approach of the phosphate backbones between the middle and side strands is ∼10 Å, which is comparable to proximities observed in the eukaryotic nucleosome (42). Moreover, the cross-links data suggest that the electrostatic repulsion at these closest-approach regions may be further mitigated by positively charged interdomain linker regions. Overall, the model points to an efficient utilization of the RNA binding capacity of the N dimer, which is required for packing the relatively large viral genome.

Of special interest are cross-links that contain an overlapping peptide pair. Such cross-links necessarily report on a direct interaction between two chains of N. We identified four sequence regions that form such cross-links (residues 100 to 102, 237 to 237, 248 to 249, and 266 to 266). These identifications are supported by well-annotated MS/MS fragmentation spectra (Dataset S5). In several cases, these identifications are supported by more than one peptide pair. Our dimer model can explain the interchain cross-links between Lys100 and Lys102 that are closer than 25 Å in several docking solutions. On the other hand, the interchain cross-links of 248 to 249 and 266 to 266 are not consistent with any model or structure. Most likely they originate from a higher-order oligomeric state of N, which we did not try to model here due to insufficient data. Finally, the interchain interaction around residue 237 is supported by several cross-links: three different peptide pairs reporting a cross-link between Lys233 and Lys237 and two different peptide pairs reporting a cross-link between Lys237 and Lys237. The multiple cross-links report on a strong interaction of the linker regions that immediately precede the two N termini of the dimerization domain. In the dimer context, these interactions occur on top of the middle RNA strand and between the two other strands bound to the RNA binding domains. This implies that additional positive charges from these linker regions are located between the strands, thereby mitigating their electrostatic repulsion. The same interaction can also serve to clamp the RNA in place.

### Targeted In Situ CLMS of Nsp1.

**Fig. 5.**
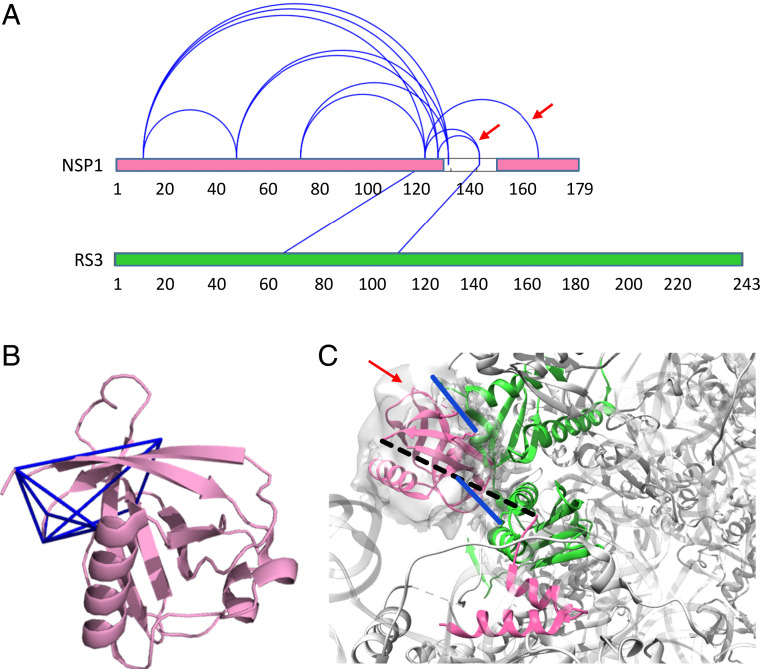
Nsp1 cross-links. (*A*) In situ cross-links identified within Nsp1 and between Nsp1 and ribosomal protein RS3. The two red arrows indicate cross-links that are not consistent with the structure of the C terminal of Nsp1 bound to the ribosome. (*B*) In situ cross-links (blue lines) within Nsp1 mapped onto the crystal structure of the N-terminal domain [PDB ID code 7k3n (56)]. (*C*) Mapping of the Nsp1-RS3 cross-links (blue) onto the cryo-EM structure of the C-terminal of Nsp1 (pink) bound to the 40S ribosomal subunit (gray) [PDB ID code 6lzw (43)]. RS3 is marked in green. The crystal structure of the N terminal is docked into the unassigned density of the structure (red arrow). The unstructured linker between the N and C domains of Nsp1 is depicted by a dashed line. One of the cross-links involves a lysine within the unstructured linker.

Nsp1 inhibits protein translation in the host cell, thereby interfering with the cellular antiviral response (24). This short protein comprises a structured N-terminal domain (residues 1 to 125) and a disordered C-terminal tail (residues 126 to 180). Two recent cryo-EM studies (43, 44) revealed the C terminal (residues 148 to 180) to bind strongly to the messenger RNA (mRNA) entry tunnel of the ribosome, thus obstructing the tunnel and inhibiting protein synthesis. The results we obtained from targeted in situ CLMS of Nsp1 fully support these findings. The main proteins that copurified with Nsp1 were components of the 40S ribosomal subunit, in particular the ribosomal S3 protein and the eukaryotic translation initiation factor 3 (eIF3) (*SI Appendix*, Fig. S4*C*). A search for cross-links among these sequences identified 12 intraprotein cross-links in Nsp1 and two cross-links between Nsp1 and RS3 (Fig. 5*A* and Dataset S4). Another three cross-links were identified within the eIF3, consistent with Nsp1 binding also to the 43S preinitiation complex (44). The estimated FDR of this set is less than 5% (*SI Appendix*, Fig. S5*D* and S6*D*). The intraprotein cross-links within Nsp1 fit well to the available crystal structure of the N-terminal domain (Fig. 5*B*). The two cross-links between Nsp1 and RS3 are in accord with the likely path of the C-terminal along the mRNA entry tunnel (Fig. 5*C*).

Three in situ cross-links are not compatible with the structure of the C terminal lodged deep within the mRNA entry tunnel (120 to 141, 125 to 141, and 120 to 164). Because the bulky N terminal cannot enter the mRNA entry tunnel, the occurrence of these cross-links implies an additional conformation of Nsp1 in which the C terminal is interacting with the N terminal. It is suggestive of an auto-inhibition mechanism for Nsp1 in which the N terminal modulates the availability of the C terminal toward the interaction with the ribosome.

## Discussion

The results establish the effectiveness of targeted in situ CLMS and integrative structure modeling to study a variety of proteins. The large number of identified cross-links allowed us to apply integrative structure modeling for domain assembly (Nsp2), oligomerization and domain assembly (N), and complex assembly (Nsp1-ribosome). Integration of domain-level models generated by deep-learning methods (AlphaFold2) with in situ CLMS data enabled ab initio structure modeling of the relatively long Nsp2 (638 amino acids). Table 1 summarizes the precisions obtained for each modeled system. Clearly, all the results presented here were greatly facilitated by the availability of high-quality models or structures for the various globular domains. This prior structural information simplified the modeling process to a combinatorial docking problem with far fewer degrees of freedom. We foresee that a rapidly growing number of biological systems will be amenable to similar modeling, as experimental and computational structure prediction approaches advance.

**Table 1.** Modeling results: the size of the cluster (number of models), the precision measured as an average rmsd between cluster members, and the fraction of satisfied cross-links

Protein	No. of models	Precision, Å	Cross-links satisfaction
Nsp2 (primary set)	62	8	43/43 (100%)
Nsp2 (primary and secondary sets)	9	1	81/83 (98%)
N dimer	100	10	13/14 (93%)

Two factors contributed significantly to the identification yield of the in situ CLMS. The first is the use of the highly hydrophobic DSS reagent for cross-linking. The hydrophobicity improves the membrane permeability, thereby allowing shortening the incubation time considerably. In our opinion, shortening the incubation times is crucial for maintaining the native cell state while minimizing the toxic effects of the cross-linker. The second factor is the utilization of the Strep tag technology that proved to be both highly effective for purification and highly compatible with CLMS. On the other hand, a major limitation of the approach employed here is that each protein is studied in isolation, without the context of the full viral infection. This simplification may also be the underlying reason for the paucity of identifications of interprotein cross-links, which would have formed if the appropriate viral interactors were present. Therefore, a future direction for targeted in situ CLMS should aim to emulate more detailed infection scenarios.

A general observation from this study is the large variation between in situ and in vitro experiments. For both Nsp2 (*SI Appendix*, Fig. S9*B*) and N (*SI Appendix*, Fig. S10) the overlap between the cross-link sets is partial, even though the underlying chemical reactivity is identical. One would expect the in vitro sets to fully contain the in situ sets, because the former are not encumbered by issues of membrane permeability. Yet, notably, a considerable number of cross-links are found only in the in situ sets. We interpret these results to indicate that in situ cross-linking probes protein states that are not occurring in vitro. These states may require certain cellular factors that are depleted upon cell lysis. Overall, the in situ/in vitro disparity highlights the importance of developing in situ techniques for the study of recalcitrant proteins. Accordingly, we believe that future in situ CLMS experiments in the context of a full viral infection would provide an even more informative picture on the functions of these proteins.

## Methods

### Cloning.

The plasmid for expression of the N protein is based on the pcDNA3.4 backbone (Thermo). Complementary DNA (cDNA) of SARS-CoV2 was generated from a clinical RNA sample (Hadassah Medical Center, Clinical Virology Laboratory, D.G.W.), using QuantaBio qscript cDNA synthesis kit, followed by amplification of the N coding region by specific primers. The amplified PCR fragment of the N coding region was subsequently cloned using Gibson assembly reaction into pcDNA3.4 backbone modified to include C-terminal Strep Tag-II (IBA). The sequence of the cloned N with C-terminal Strep tag was verified using Sanger sequencing. The sequence is identical to the canonical N sequence (UniProt ID P0DTC9). Plasmids for expression of Nsp1 and Nsp2 with a Strep-tag were kindly provided by the Krogan laboratory (45). All plasmids were amplified under ampicillin selection in Top10 cells (Invitrogen) and purified by PureLink (Invitrogen).

### Cell Culture and Transfection.

Human embryonic kidney cells 293 (HEK293; ATCC) were cultured (Dulbecco’s modified Eagle’s medium high-glucose, 10% fetal bovine serum) at 37 °C, 5% CO_2_, and high humidity. Three days prior to the transfection, the cells were plated in a 10-cm plate at an initial density of 2.25 × 10^6^ cells per plate. Expression plasmids (Nsp1/Nsp2/N) and PEI (260008-5; Polysciences) were separately diluted in Opti-MEM1 (31985-047; Gibco) and mixtures were incubated at room temperature for 25 min to allow polyplex formation prior to its addition to the cell culture. The mixture was added dropwise onto the cells. The plates were washed and fed with fresh medium 24 h posttransfection. The cells were dissociated 40 h after transfection by application of Dulbecco’s phosphate-buffered saline without calcium and magnesium (D-PBS) supplemented with 10 mM ethylenediaminetetraacetic acid (EDTA) for 5 min at 37 °C. The cells were pelleted and transferred into a 1.7-mL tube. Pelleting of intact cells was always carried out by centrifugation at 200 × *g* for 3 min at either room temperature (for buffers at 37 °C) or 4 °C (for ice-cold buffers).

### In Situ Cross-Linking.

The in situ cross-linking followed a recently described protocol (46). Briefly, the cell pellet was resuspended in cross-linking buffer, which was warm D-PBS supplemented with either 0.3% formaldehyde or 10 mM DSS. Note that the mixing of DSS with D-PBS resulted immediately in a cloudy solution because of the poor solubility of DSS in aqueous buffers. The rationale for the value of 10 mM was to ensure that the DSS reservoir in the buffer is not depleted during the cross-linking incubation. The cells were incubated with the cross-linker for 20 min at 37 °C under constant gentle agitation to ensure that a cell pellet did not form. The cells were pelleted and the cross-linking buffer was replaced with ice-cold quenching buffer (50 mM Tris⋅HCl, pH 7.5, 150 mM NaCl, and 1 mM EDTA) to inactivate excess cross-linker around the intact cells. Quenching proceeded at 4 °C for 10 min under gentle agitation. The cells were pelleted and the quenching buffer was removed.

### Affinity Purification.

The cells were resuspended in 600 μL of lysis buffer (50 mM Tris⋅HCl, pH 7.5, 150 mM NaCl, 1 mM EDTA, 0.5% Triton X-100, and 1% protease inhibitor mixture (P8340; Sigma) and lysed by sonication (46). Avidin (A9275; Sigma) was added to the cleared lysate to a final concentration of 0.11 mg/mL and incubated at 4 °C for 1 h. The supernatant was then incubated at 4 °C for 2 h with 10 μL of StrepTactin resin (Sepharose High Performance, Cytiva). The lysate was removed, and the resin was manually washed three times with 1 mL of wash buffer (50 mM Tris⋅HCl, pH 7.5, 150 mM NaCl, and 1 mM EDTA). Throughout the washing steps, the resin was brought to the bottom of the tube by centrifugation at 200 × *g* for 45 s. The overall duration of the washing step was 6 min. To elute the protein, the beads were covered with a wash buffer supplemented with 10 mM biotin for 30 min with occasional gentle mixing. The supernatant was collected and prepared for MS.

### In Vitro Cross-Linking of Nsp2.

For in vitro cross-linking, the dissociated cells were immediately resuspended in the Hepes lysis buffer and lysed as described above. The affinity purification was modified to use Hepes wash buffer (Hepes, pH 8.0, and 200 mM NaCl, no detergent added) and Hepes elution buffer (Hepes wash buffer with 10 mM biotin). For testing the structural effect of the cation environment, the Hepes buffer was supplemented throughout all the steps with either 50 μM ZnCl2, 50 μM MgCl2, or no cation addition. For BS3 [bis(sulfosuccinimidyl)suberate] cross-linking, the eluted protein was incubated with either 0.5 or 1 mM BS3 at 30 °C for 30 min, quenched with 20 mM ammonium bicarbonate for 10 min, and then prepared for MS.

### Preparation of Samples for MS.

The digestion protocol was recently described (46). Based on ultraviolet absorbance measurements, the final amounts per experiment of peptides in the tryptic digest were 20 μg, 2 μg, and 200 ng for N, Nsp2, and Nsp1, respectively. Despite the low amount, we attempted to enrich for cross-linked peptides in the case of Nsp2. To that end, peptides from two different purifications were enriched by either strong cation exchange chromatography (47) or size-exclusion chromatography (48). The enrichments led to the identification of three new cross-links (out of 53) over the standard mass spectrometric analysis of the full digest. We conclude that the common enrichment techniques are not effective for protein samples of such low amounts.

### MS.

The samples were analyzed by a 120-min 0-to-40% acetonitrile gradient on a liquid chromatography system coupled to a Q-Exactive HF mass spectrometer. The analytical column was an EasySpray 25 cm heated to 40 °C. The method parameters of the run were as follows: Data-Dependent Acquisition; Full MS resolution 70,000 ; MS1 AGC target 1e6; MS1 Maximum IT 200 ms; Scan range 450 to 1,800; dd-MS/MS resolution 35,000; MS/MS AGC target 2e5; MS2 Maximum IT 600 ms; Loop count Top 12; Isolation window 1.1; Fixed first mass 130; MS2 Minimum AGC target 800; Peptide match - off; Exclude isotope - on; Dynamic exclusion 45 seconds. Each cross-linked sample was measured twice in two different HCD energies (NCE): 26, and stepped 25, 30, and 35. All cross-linked samples were measured with the following charge exclusion: unassigned,1,2,3,8,>8. Proteomics samples were measured with the following charge exclusion: unassigned,1,8,>8.

### Proteomics Analysis of Interacting Proteins.

Human proteins that interact with the bait proteins were identified by comparing the protein content between purifications from transfected and untransfected cells. Label-free quantification (LFQ) was performed with MaxQuant 1.5 (49) using the default parameters. The sequence database comprised all human proteins (downloaded from UniProt) augmented with the sequences of three SARS-CoV-2 proteins: Nsp1, Nsp2, and N protein. The “proteinGroups.txt” output file was loaded to Perseus (50). Reverse proteins and contaminations were filtered out, the data transformed to logarithmic scale, and samples grouped according to replicates. Only proteins identified by more than two peptides were considered. For missing LFQ intensities, values were imputed from a normal distribution. The confidence curves were determined by a two-sample test with a permutation-based FDR of 0.5% and “s0” (minimal fold change) value of 2. The Volcano plots present the proteins in the “t-test difference” vs. “-Log t-test p-value” coordinate system.

### Identification of Cross-Links.

The identification of cross-links followed the procedure described recently (46). The sequence databases for each target protein included all the proteins above the confidence line in the volcano plots (*SI Appendix*, Fig. S4). The FDR was estimated from decoy-based analysis, which repeated the identification analysis 20 times with an erroneous cross-linker mass of 138.0681*N/138 Da, where *n* = 160, 161, 162, … 179. This led to bogus identifications with fragmentation scores that were generally much lower than the scores obtained with the correct cross-linker mass (see histograms in *SI Appendix*, Fig. S6). For the identification of true cross-links, we set the threshold on the fragmentation score according to the desired FDR value. For example, a threshold of 0.65 on the fragmentation score of the Nsp2 dataset gave 53 cross-links above the threshold in the true analysis and a median of 1 cross-link in a typical decoy run (*SI Appendix*, Fig. S5). We therefore estimate the corresponding FDR to be about 1 in 53, or ∼2%. The final thresholds used: Nsp2 Primary Set – 0.65, Nsp2 Secondary Set – 0.9, Nsp2 in vitro Set – 0.65, N – 0.7, Nsp1 – 0.65.

### Domain Assembly via Pairwise Docking.

The input to the domain assembly problem consists of a set of structural models and a list of cross-links. The goal is to predict an assembly with good complementarity between the domains and consistency with the input cross-links. We use CombDock (31), a combinatorial docking algorithm, which was modified to support cross-linking data (33). First, pairwise docking is applied on each pair of input structures to generate a set of docked configurations (Step 1). Second, combinatorial optimization is used to combine different subsets of the configurations from pairwise docking to generate clash-free complex models consistent with the cross-links and chain connectivity (Step 2). A cross-link is considered satisfied if the distance between the Cа atoms of the cross-linked residues is below a specified threshold. Here we used a threshold of 25 Å and 20 Å for DSS and formaldehyde cross-links, respectively.

A benchmark of this algorithm is shown in *SI Appendix*, Fig. S11, in which we model the full holoenzyme of ribonucleotide reductase class 1b from *Mycoplasma pneumoniae* by docking its two heterodimers. The inputs for the run were based on a recent set of in situ cross-links (17) from *M. pneumoniae* and homology models of each dimer from other bacterial species. We were able to obtain a model that satisfied 181/212 (85%) of the cross-links and had a ligand-centered rmsd of 10.8 Å to a homolog (PDB ID code 2BQ1).

### Step 1: All-Pairs Docking.

We used PatchDock to generate pairs of docked configurations (32). PatchDock employs an efficient rigid docking algorithm that maximizes geometric shape complementarity. Protein flexibility is accounted for by a geometric shape complementarity scoring function, which allows a small amount of steric clashes at the interface. Only domain pairs with at least one cross-link between them were structurally docked at this stage. The PatchDock scoring function was augmented by restraints derived from the cross-linking data. Each docking configuration is represented by a transformation (three rotational and three translational parameters) and we keep the K = 1,000 best scoring transformations for each pair of domains.

### Step 2: Combinatorial Optimization.

Two basic principles are used by the algorithm: a hierarchical construction of the assembly and a greedy selection of subcomplexes. The input comprises the pair-wise docking of Step 1 (subcomplexes of size 2). At each step, the algorithm generates subcomplexes with n subunits by connecting two subcomplexes of smaller size. Only valid subcomplexes are retained at each step. Valid subcomplexes do not contain steric clashes and satisfy distance constraints (chain connectivity) and restraints (cross-links). Searching the entire space is impractical, even for relatively small K (number of models per pair) and N (number of domains), due to computer speed and memory limitations. Therefore, the algorithm performs a greedy selection of subcomplexes by keeping only the D = 1,000 best-scoring models at each step. The final models are clustered using rmsd clustering with a cutoff of 4 Å. The final best-scoring models are selected based on the cross-links satisfaction and cluster size. The model precision is calculated as the average Cα rmsd between the best-scoring models.

Pairwise docking was applied to dock the RNA binding domain of N (PDB ID code 7act) to the dimerization domain (PDB ID code 6zco). Combinatorial optimization was performed for assembly of Nsp2 domains.

### MD Simulations.

MD simulations were performed on the dimerization domain of the N protein model with docked RNA using GROMACS 2020 software (51) and the PARMBSC1 force field (52). Any steric clashes between the docked RNA and protein were resolved by removing nucleotide bases, leaving a poly-U hexameric RNA fragment. Then, the protein–RNA complex was solvated with a simple point charge water model with a self-energy polarization correction term (SPC/E) (53), and the system charge was neutralized with the addition of Cl^−^ ions. In order to ensure appropriate initial geometry, a steepest-descent energy minimization MD run was allowed to run until convergence at Fmax < 1,000 kJ/(mol·nm). Position restraints with k = 1,000 kJ/(mol·nm^2^) are then applied to the protein and RNA heavy atoms to allow the water and ions to equilibrate around the protein in two-steps. The first equilibration step is conducted at a constant number of atoms, volume, and temperature (NVT) of 300 K. The second equilibration step is conducted at a constant number of atoms, pressure of 1 bar, and temperature of 300 K (NPT). These equilibrations have a time step of 2 fs and last for 100 ps. Once the system is equilibrated at 300 K and 1 bar, a production simulation of 100 ns with a time step of 2 fs provides 10,000 MD simulation frames at intervals of 10 ps.

## Supplementary Material

Supplementary File

Supplementary File

Supplementary File

Supplementary File

Supplementary File

Supplementary File

Supplementary File

Supplementary File

Supplementary File

## Data Availability

The mass spectrometry data have been deposited to the ProteomeXchange Consortium via the PRIDE (54) partner repository. The cross-link information is compiled with the dataset identifier PXD023487. The proteomics information is compiled with the dataset identifier PXD023542. Atom coordinates and modeling parameters have been deposited in PDB-Dev:  Accession codes 536 (Nsp2) and 537 (N).
