# Supplementary material for: Targeted in situ cross-linking mass spectrometry and integrative modeling reveal the architectures of three proteins from SARS-CoV-2
Source: Proc Natl Acad Sci U S A. 2021 Aug 9;118(34):e2103554118. doi: 10.1073/pnas.2103554118 (PMC8403911; doi:10.1073/pnas.2103554118)
Supplement: Supplementary File [file pnas.2103554118.sapp.pdf]

PNAS
www.pnas.org

Supplementary Information for

Targeted in situ cross-linking mass spectrometry and integrative modeling reveal the architectures of three proteins from SARS-CoV-2.

Moriya Slavin, Joanna Zamel, Keren Zohar, Siona Eliyahu, Merav Braitbard, Esther Brielle, Leah Baraz, Miri Stolovich-Rain, Ahuva Friedman, Dana G Wolf, Alexander Rouvinski, Michal Linial, Dina Schneidman-Duhovny, Nir Kalisman

Email: michall@mail.huji.ac.il (M.L.) ; dina.schneidman@mail.huji.ac.il (D.S.) ; nirka@mail.huji.ac.il (N.K.)

This PDF file includes:

Figures S1 to S11
Legends for Movie S1
Legends for Datasets S1 and S7
SI References

Other supplementary materials for this manuscript include the following:

Movie S1 (MOV file)
Datasets S1 to S4 (XLSX files)
Dataset S5 (PDF file)
Datasets S6 and S7 (TXT files)

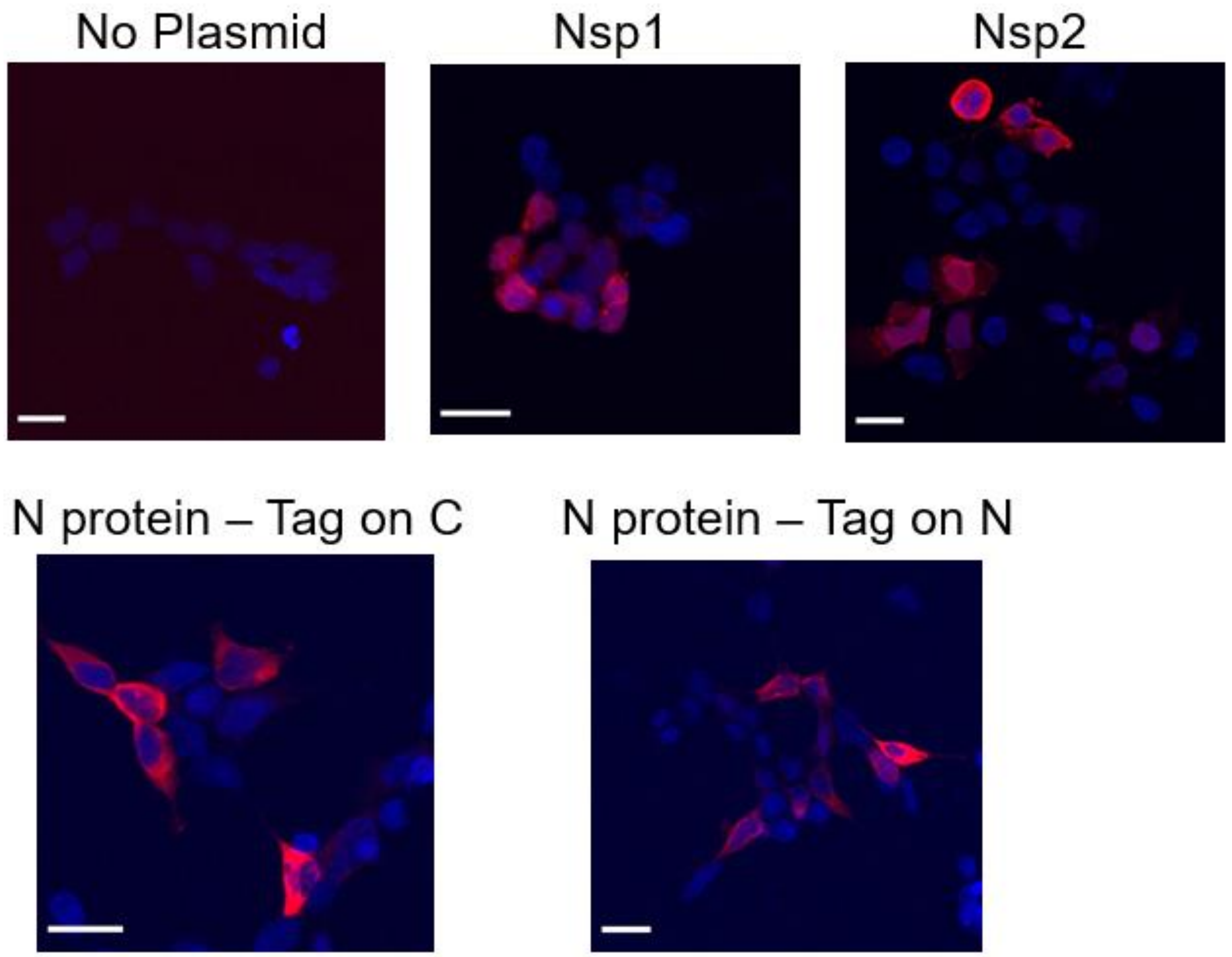

Fig. S1. Expressed proteins do not show signs of aggregation as visualized by fluorescence microscopy. Red - Staining against the Strep-tag by fluorescent streptactin. Blue - DAPI staining. Expression of the N protein was tested by two different plasmids with the Strep-tag fused to the protein at either terminal. Scale bar - 20 μm.

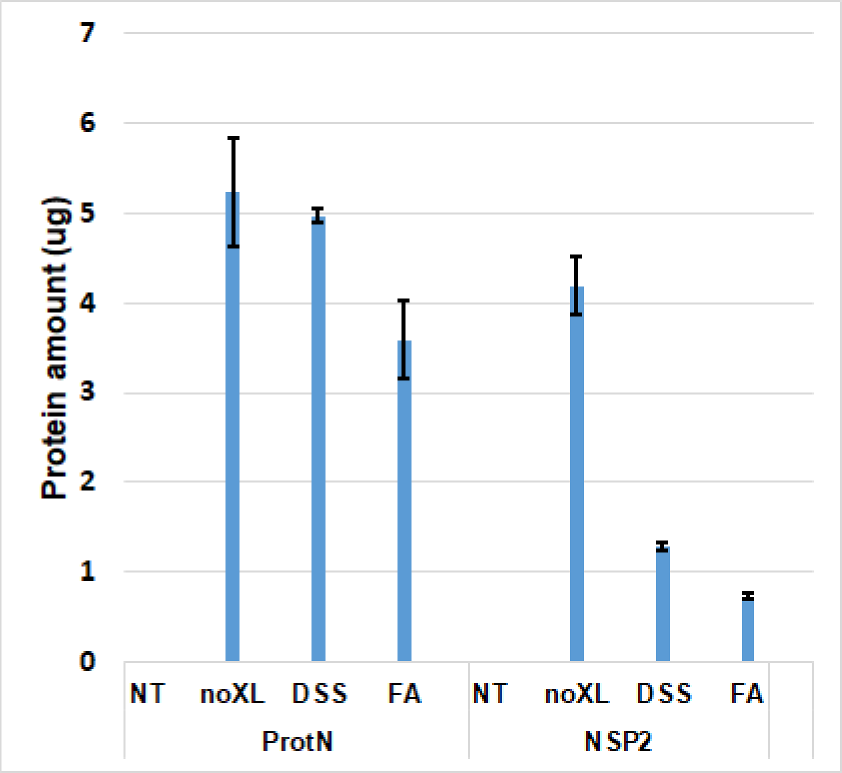

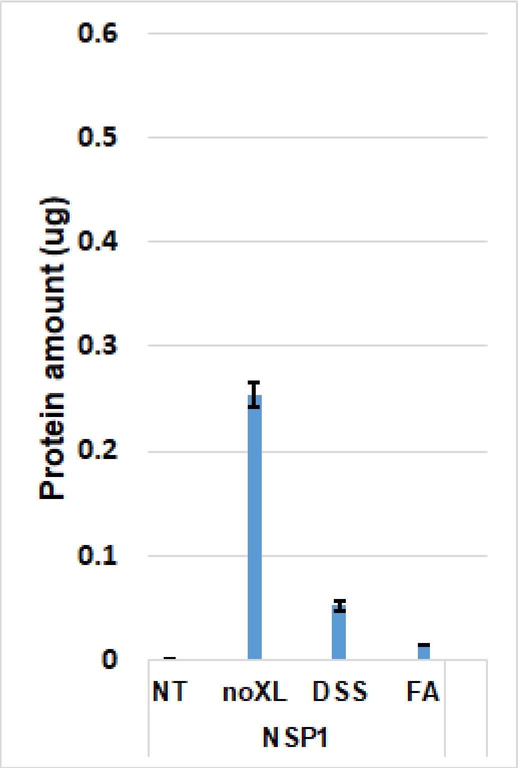

Fig. S2. Typical Strep-tag purification yields from a single 10 cm plate of cells. The absolute protein amounts were calibrated based on spiking of the eluted protein with 0.25 µg of bovine serum albumin prior to digestion. The averages and standard deviations are calculated from two experimental repeats. NT - cells were not transfected with the plasmids (control). NoXL - cells were transfected with the plasmids, but no cross-linker was applied. DSS - In situ cross-linking of the cells with DSS. FA - In situ cross-linking of the cells with formaldehyde. The lower yields of DSS and FA purifications of NSP1 and NSP2 are most likely the result of their interactions with membrane proteins that precipitate before the sample is digested.

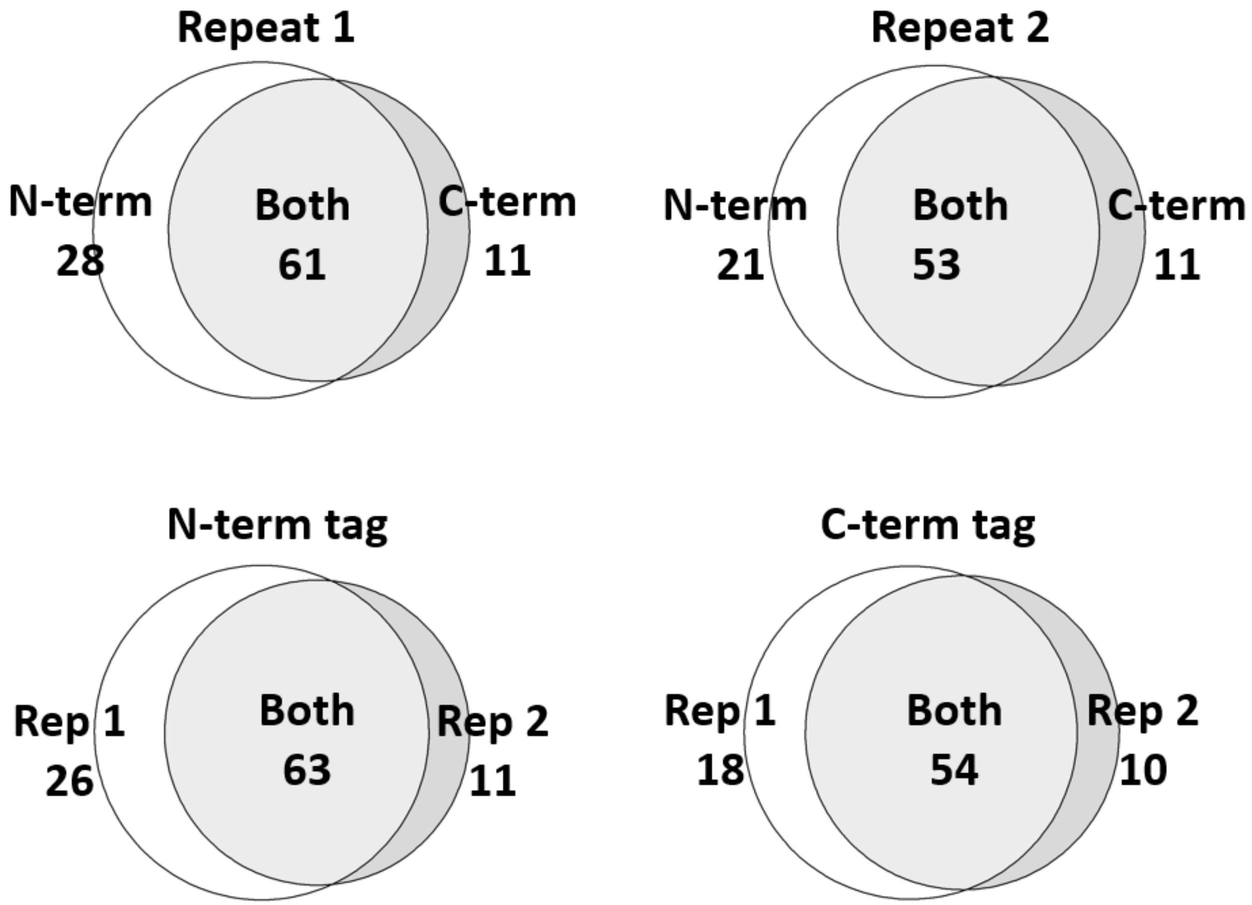

Fig. S3. The Strep-tag does not interfere with the structure of the N protein. The in situ CLMS protocol (DSS cross-linking) was repeated for two different plasmids: one with the Strep tag at the N-terminal of the N protein, and the other with the Strep tag at the C-terminal of the N protein. The sets of identified cross-links from both samples overlap significantly (top diagrams), thereby indicating that the structures are nearly identical. To show that this degree of overlap essentially reports on the same structure, we also made two technical repeats of each sample (bottom diagrams). Technical repeats are two successive mass spectrometry measurements from the same sample vial.

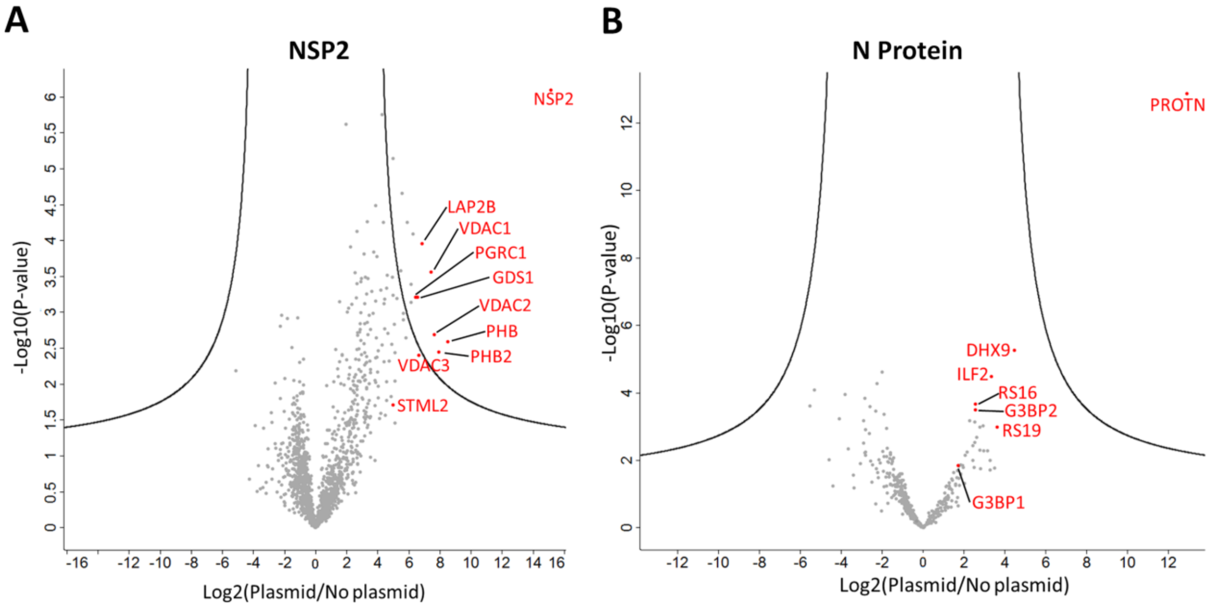

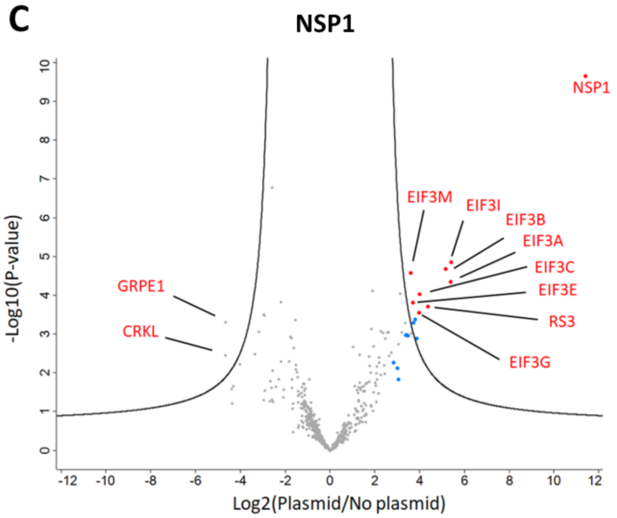

Fig. S4. Volcano plots quantifying the protein compositions of purifications from transfected vs. untransfected cells. FDR thresholds of 0.5% are marked by the black curves. **(A)** Nsp2 transfection. Components of the Prohibitin complex (PHB, VDAC, and STML2) co-elute in significant amounts. **(B)** N protein transfection. The N protein does not appear to have a specific human protein interactor **(C)** Nsp1 transfection. Components of the 40S ribosome co-eluted with Nsp1. Interestingly, the CCT chaperonin was abundant in the Nsp1 elution (blue dots mark the CCT subunits).

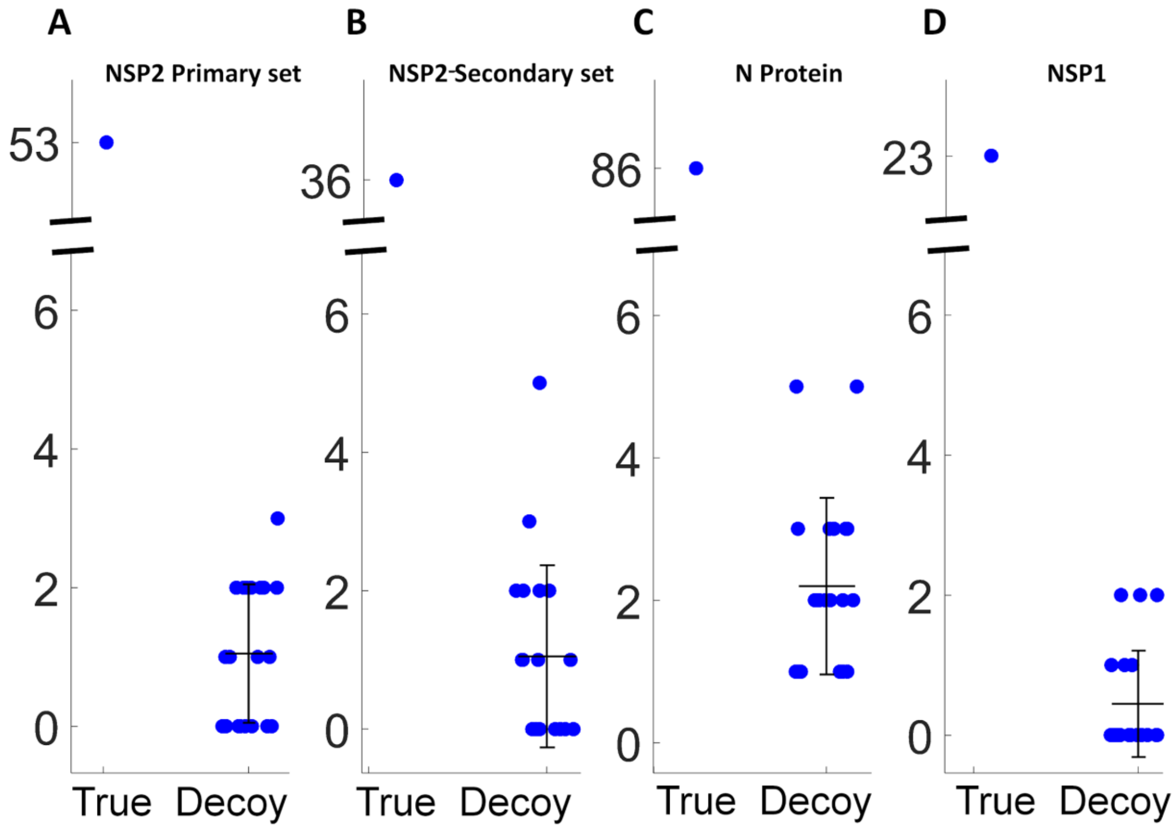

Fig. S5. Estimation of the false-detection rate (FDR) for the DSS cross-link sets. The identification analysis was repeated 20 times with an erroneous cross-linker mass that differed from the true mass (138.0681 Da) by 20 to 39 Da. Each blue dot represents the number of identified cross-links from either a true or decoy analysis. The mean and standard deviation of each decoy set is shown. The FDR for each set was the ratio of the median value of the decoys to the number of true identifications.

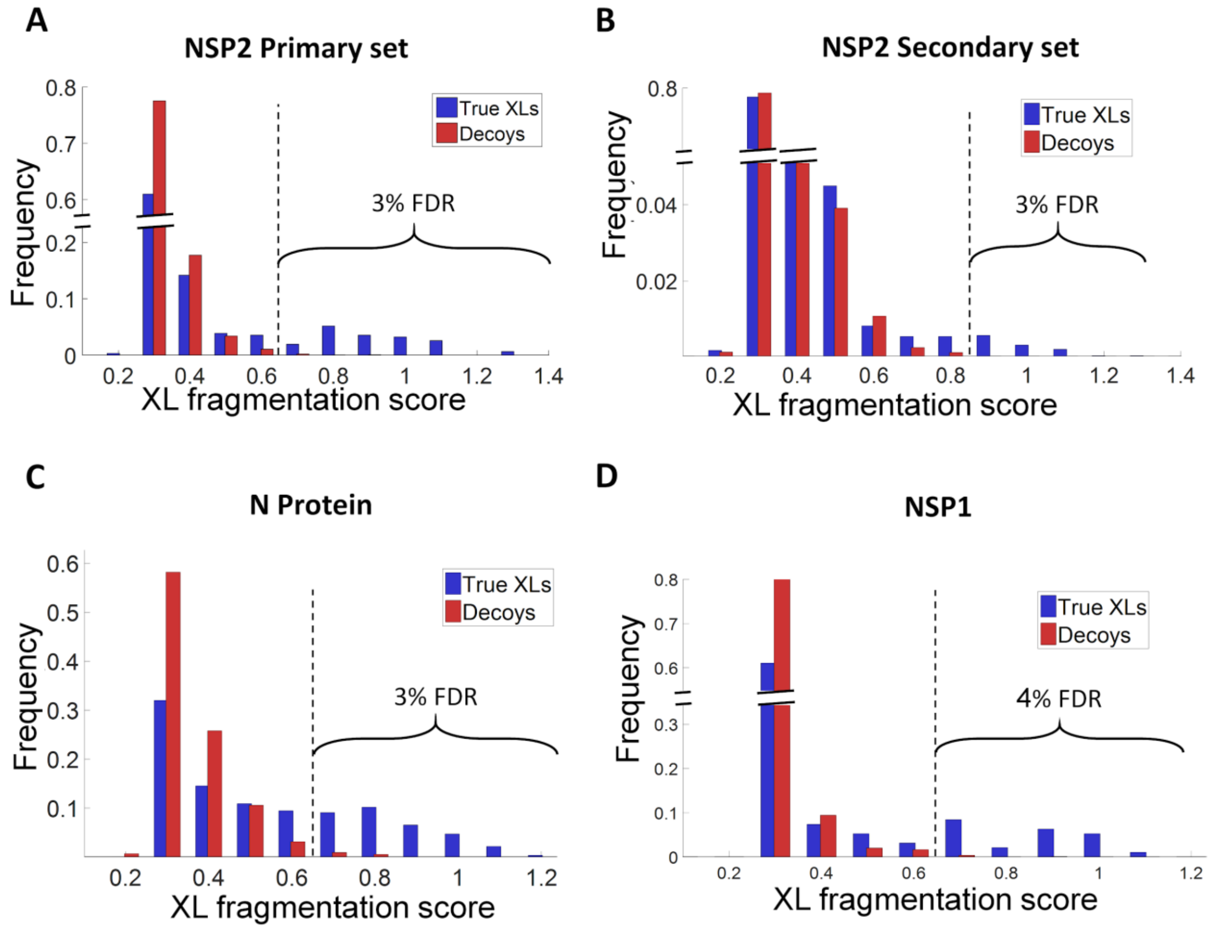

Fig. S6. Distributions of the XL fragmentation score (number of MS/MS fragments divided by the total length of the peptides). For each cross-link set, we report only the cross-links above a certain score threshold that is marked by a dashed line. **(A)** For the primary set of Nsp2 the threshold is 0.65, which sets an FDR of ~3%. **(B)** For the secondary set of Nsp2 the threshold is 0.9 **(C)** For the N protein the threshold is 0.7 **(D)** For Nsp1 the threshold is 0.65

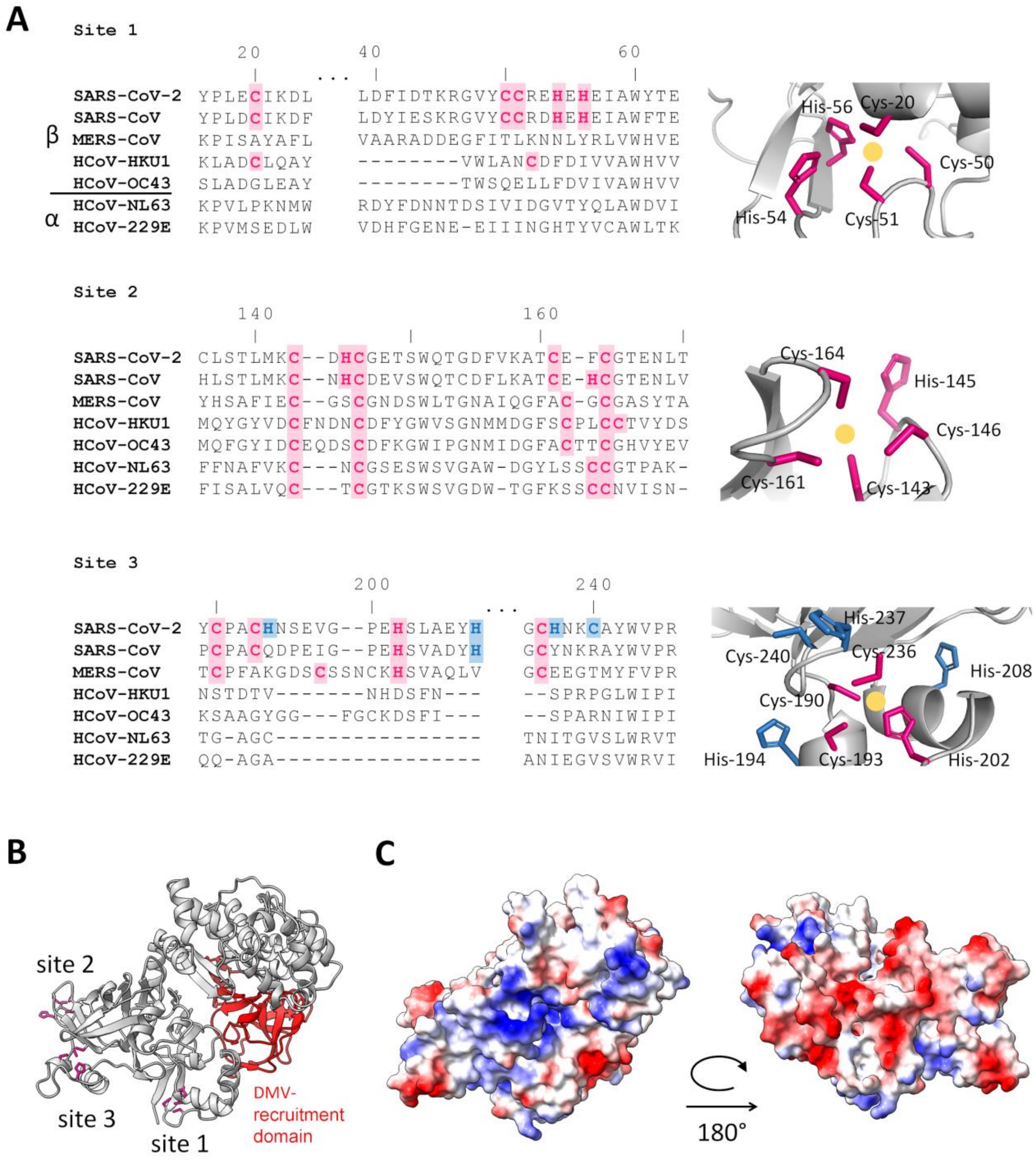

Fig. S7. Functional annotation of the Nsp2 model. (**A**) Three putative metal binding sites were identified in the model. Site 2 is conserved in all coronaviruses, while sites 1 and 3 are only occurring in the SARS subfamily. Sidechains of highlighted residues are shown in stick representation on the right. A gold circle marks the putative location of the metal ion. Zinc is the most likely substrate based on structural similarity. In SARS-CoV-2 only, an incomplete site is apparent next to site 3 (blue highlight). (**B)** The three sites are located at the opposite end of the model relative to the domain that recruits the Nsp2 to the replication-transcription complex(27). (**C)** Electrostatics analysis of the surface of the full model reveals two sides of opposite charges. The left view is the same as in Panel B. The large acidic patch in the right view is conserved across human coronaviruses (**Figure S8**).

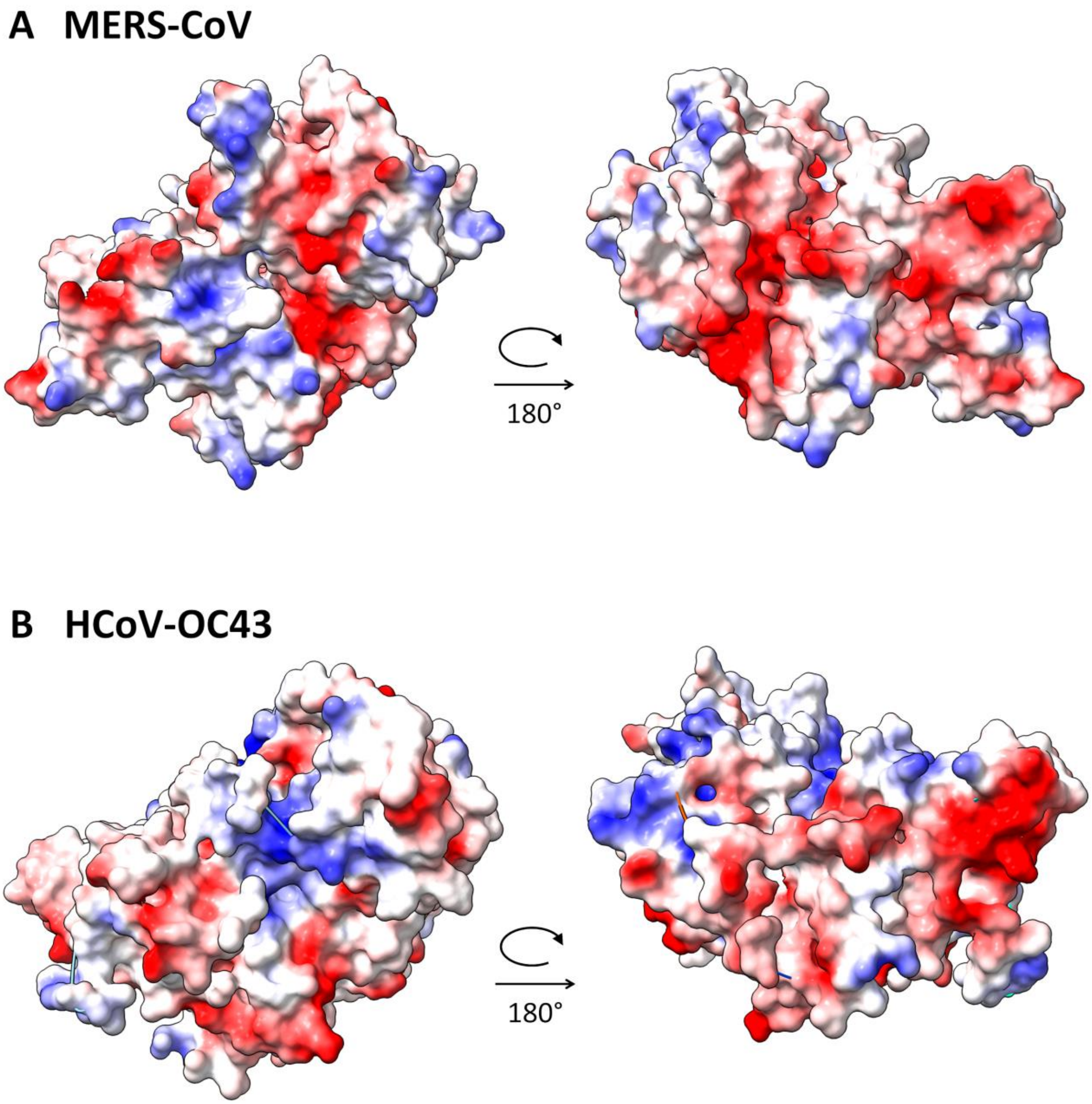

Fig. S8. Electrostatic surfaces of homology models of Nsp2 sequences of MERS and OC43 that are based on our integrative model as template. The large acidic patch (right views) is conserved across human coronaviruses.

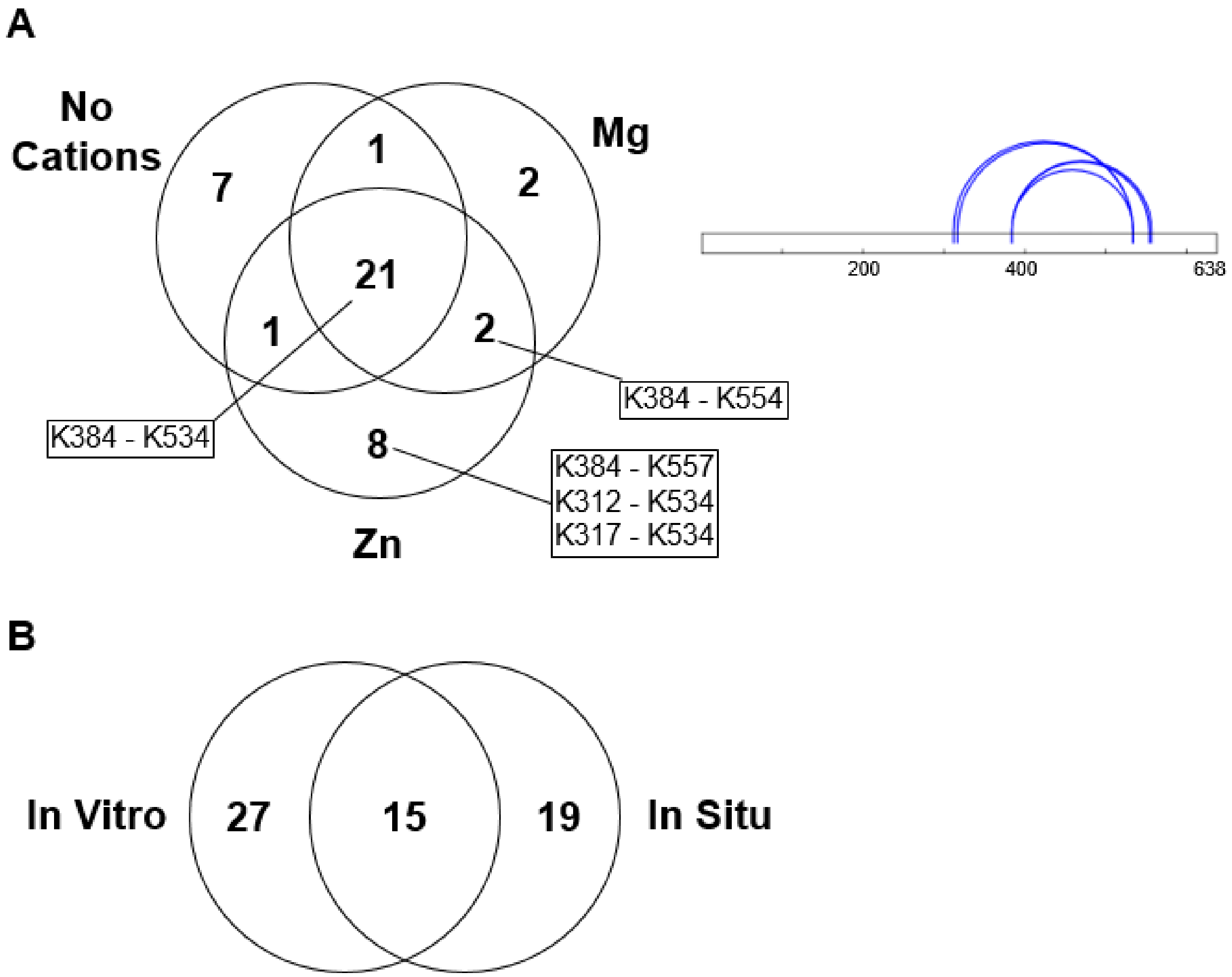

Fig. S9. In vitro CLMS of Nsp2 under different cation environments. (A) Purified Nsp2 was cross-linked by BS3 under three different cation environments: 50 μM Zn, 50 μM Mg, and No Cations. The overlap between the cross-links in the three sets is shown in the Venn diagram. Long-range cross-links that are only consistent with the closed conformation are shown in text boxes and as arcs on the right. (B) Overlap between the in situ set and a non-redundant unification of the three in vitro cation sets.

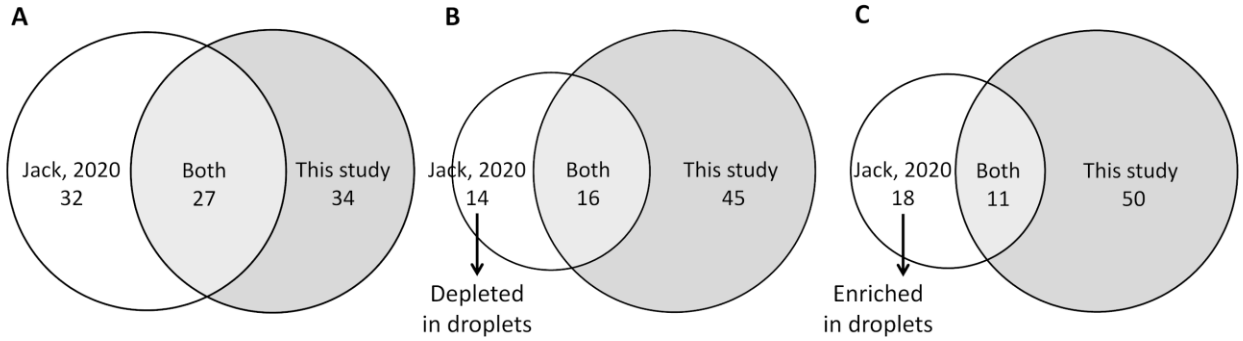

Fig. S10. Overlap of N protein cross-link sets obtained under *in vitro* conditions (Jack *et al.*[1], white) or *in situ* (this study, grey). Jack *et al*.[1] cross-linked the N protein under two conditions – soluble N protein (at high salt concentration) and phase separated N protein in droplets (at low salt concentration). They report **(A)** 59 cross-links overall, **(B)** 30 cross-links that are depleted in the phase separated condition, and **(C)** 29 cross-links that are enriched in the phase separated condition. The *in situ* cross-links seem to originate from both conditions.

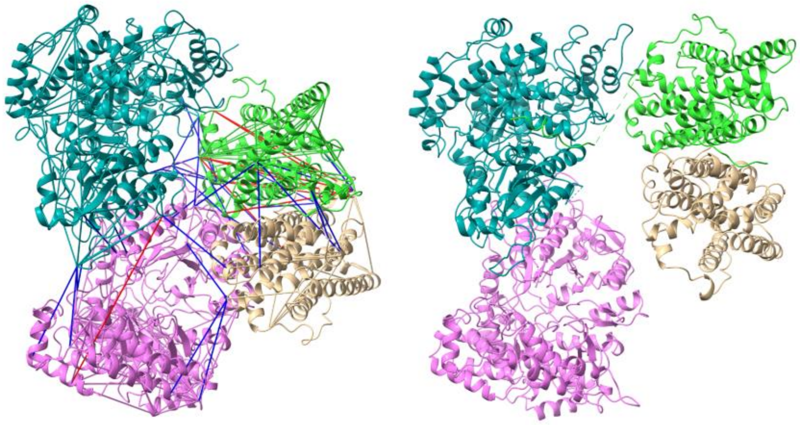

Fig. S11. Proof-of-concept model based on domain docking (template PDB 2BQ1) and in situ cross-linking (left) vs. x-ray structure of the ribonucleotide reductase homolog (right). The model satisfies 181/212 (85%) of the cross-links and is more compact compared to the homologous structure.

Movie S1. Morphing between the open and closed conformations of Nsp2. (Separate MOV file).

Dataset S1. Quantification of the 10 most abundant proteins from each purification condition shown in Figure S2. (Separate XLSX file).

Dataset S2. All cross-link sets pertaining to Nsp2. Each tab shows a different set. (Separate XLSX file).

Dataset S3. All cross-link sets pertaining to N protein. Each tab shows a different set. (Separate XLSX file).

Dataset S4. All cross-link sets pertaining to Nsp1. Each tab shows a different set. (Separate XLSX file).

Dataset S5. Annotated MS/MS spectra that report on inter-subunits cross-links of N protein. (Separate PDF file).

Dataset S6. Atom coordinates (in PDB format) for the Nsp2 model. (Separate TXT file).

Dataset S7. Atom coordinates (in PDB format) for the N dimer model. (Separate TXT file).

SI References

1. A. Jack, et al., SARS CoV-2 nucleocapsid protein forms condensates with viral genomic RNA. bioRxiv (2020) https:/doi.org/10.1101/2020.09.14.295824
