# Supplementary material for: Targeted in situ cross-linking mass spectrometry and integrative modeling reveal the architectures of three proteins from SARS-CoV-2
Source: Proc Natl Acad Sci U S A. 2021 Aug 9;118(34):e2103554118. doi: 10.1073/pnas.2103554118 (PMC8403911; doi:10.1073/pnas.2103554118)
Supplement: Supplementary File [file pnas.2103554118.sd05.pdf]

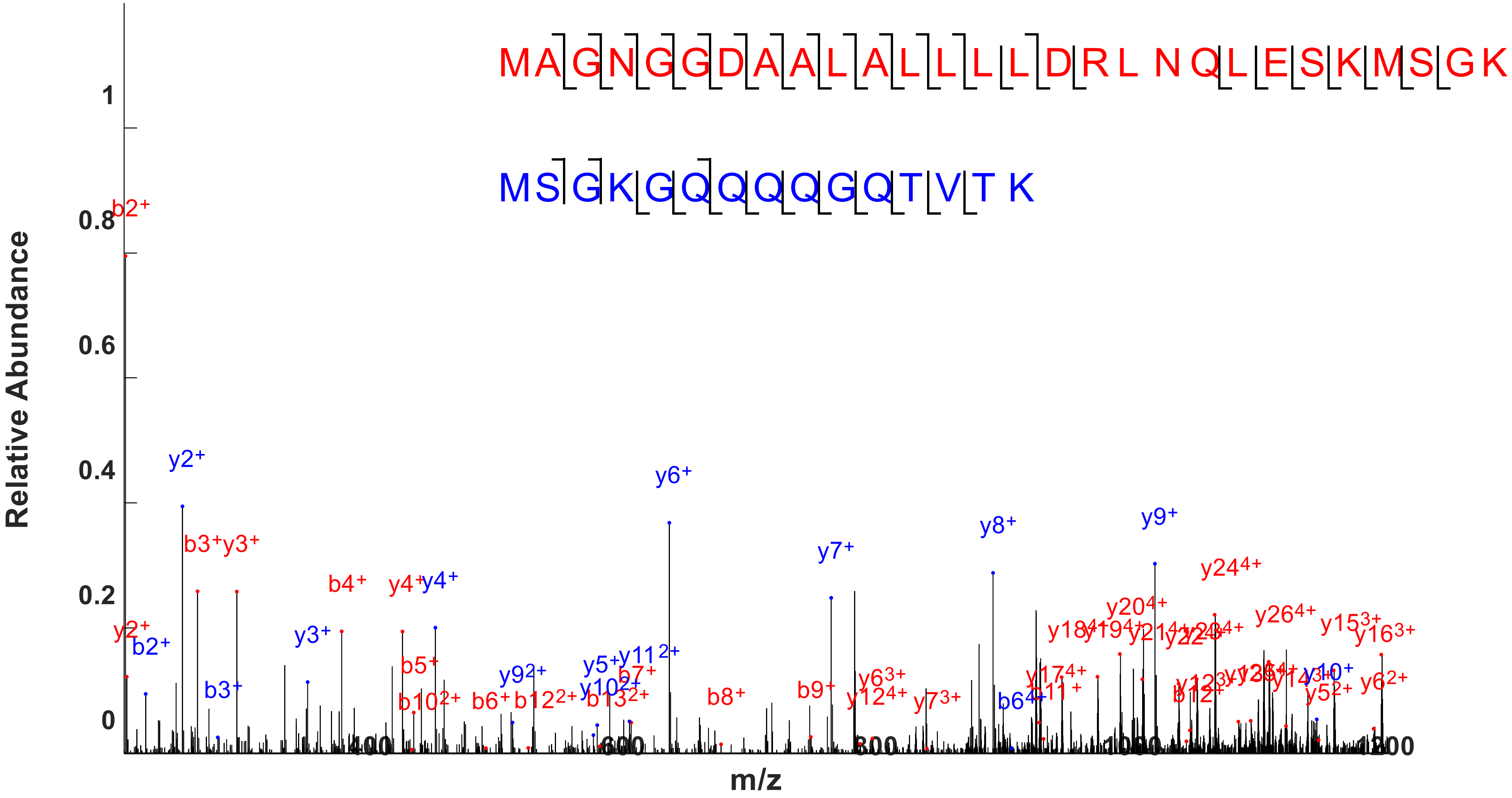

Protein N dimer cross link (1) K233 – K237.

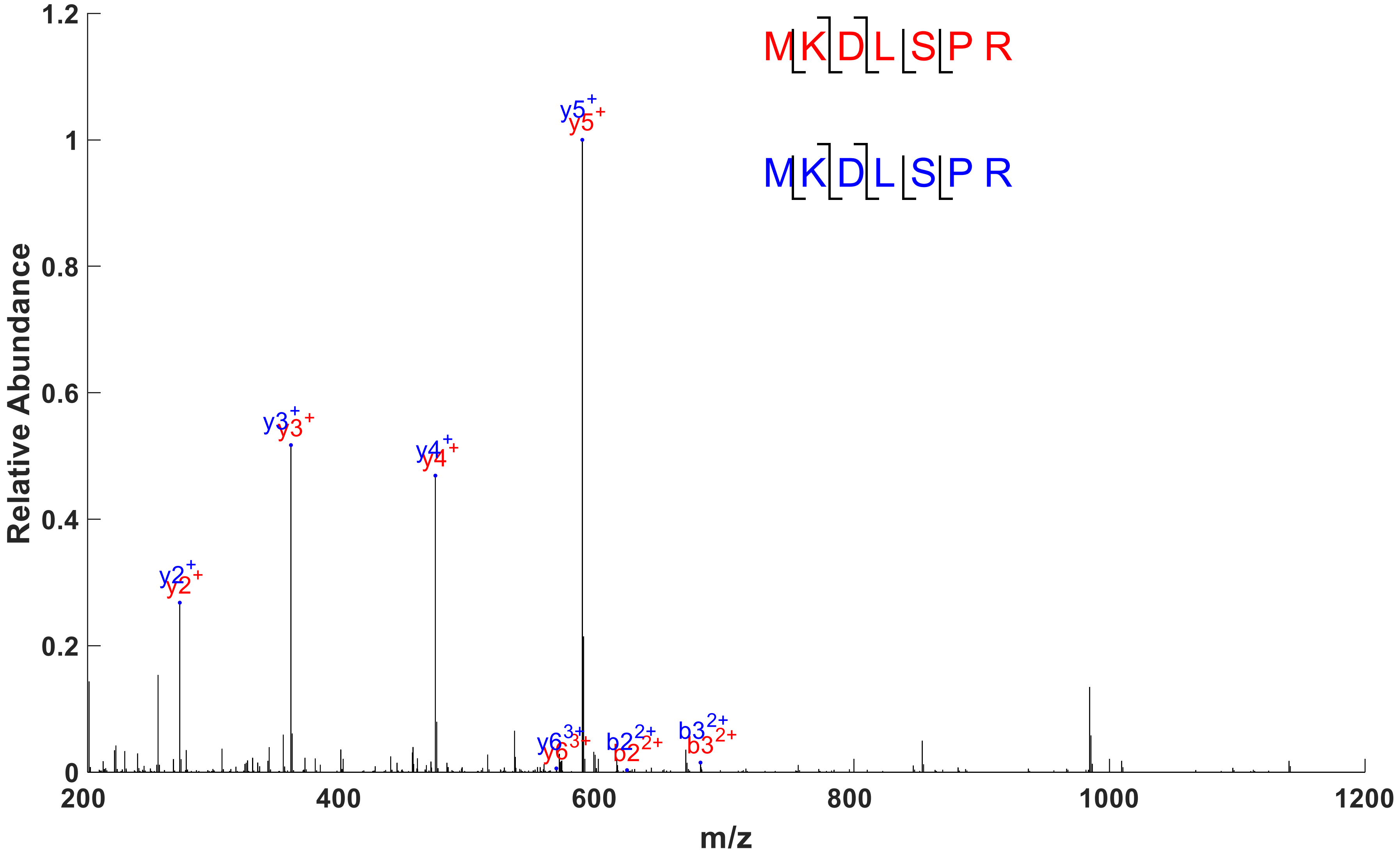

Protein N dimer cross link (2) K102 – K102.

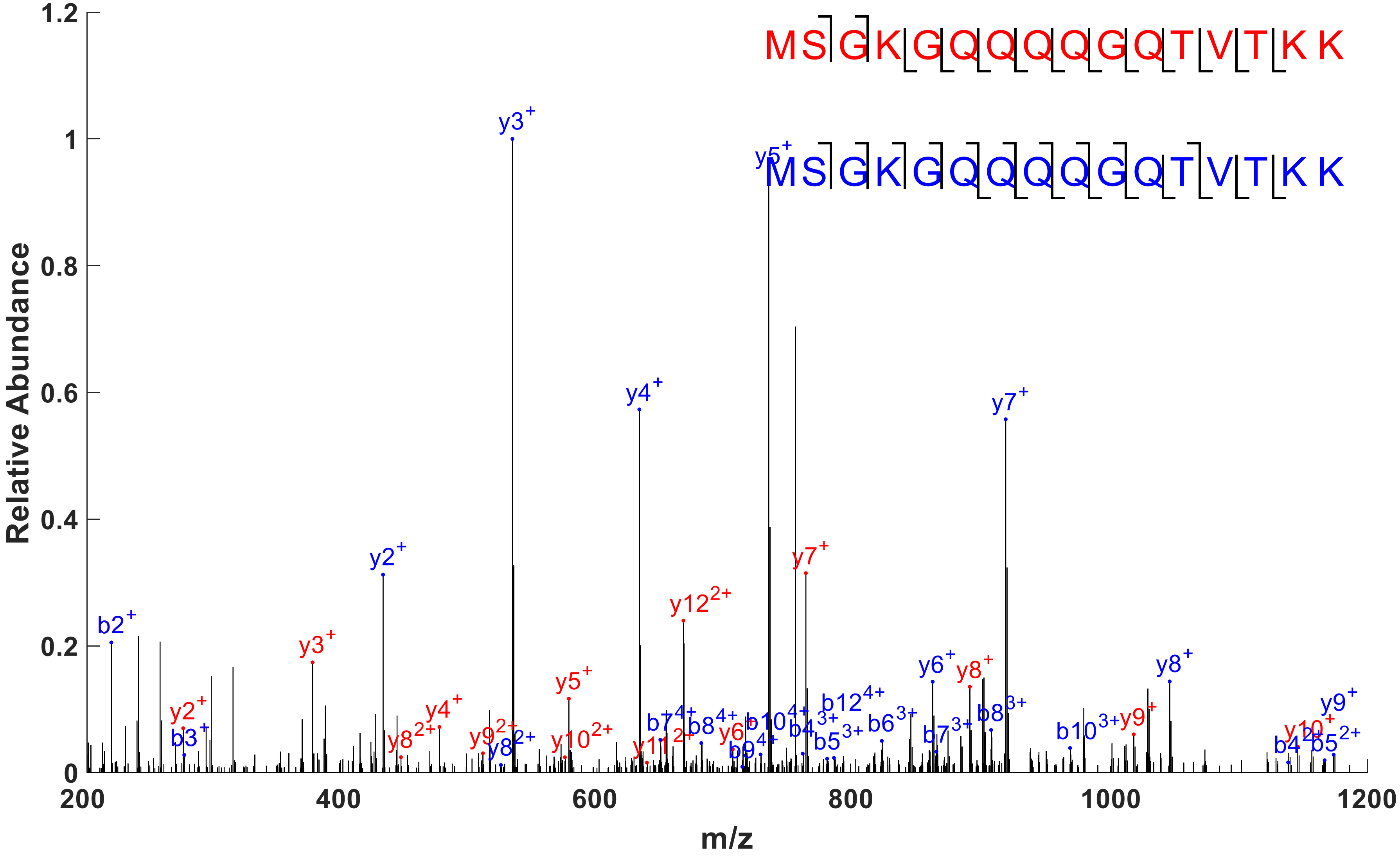

Protein N dimer cross link (3) K237 – K237.

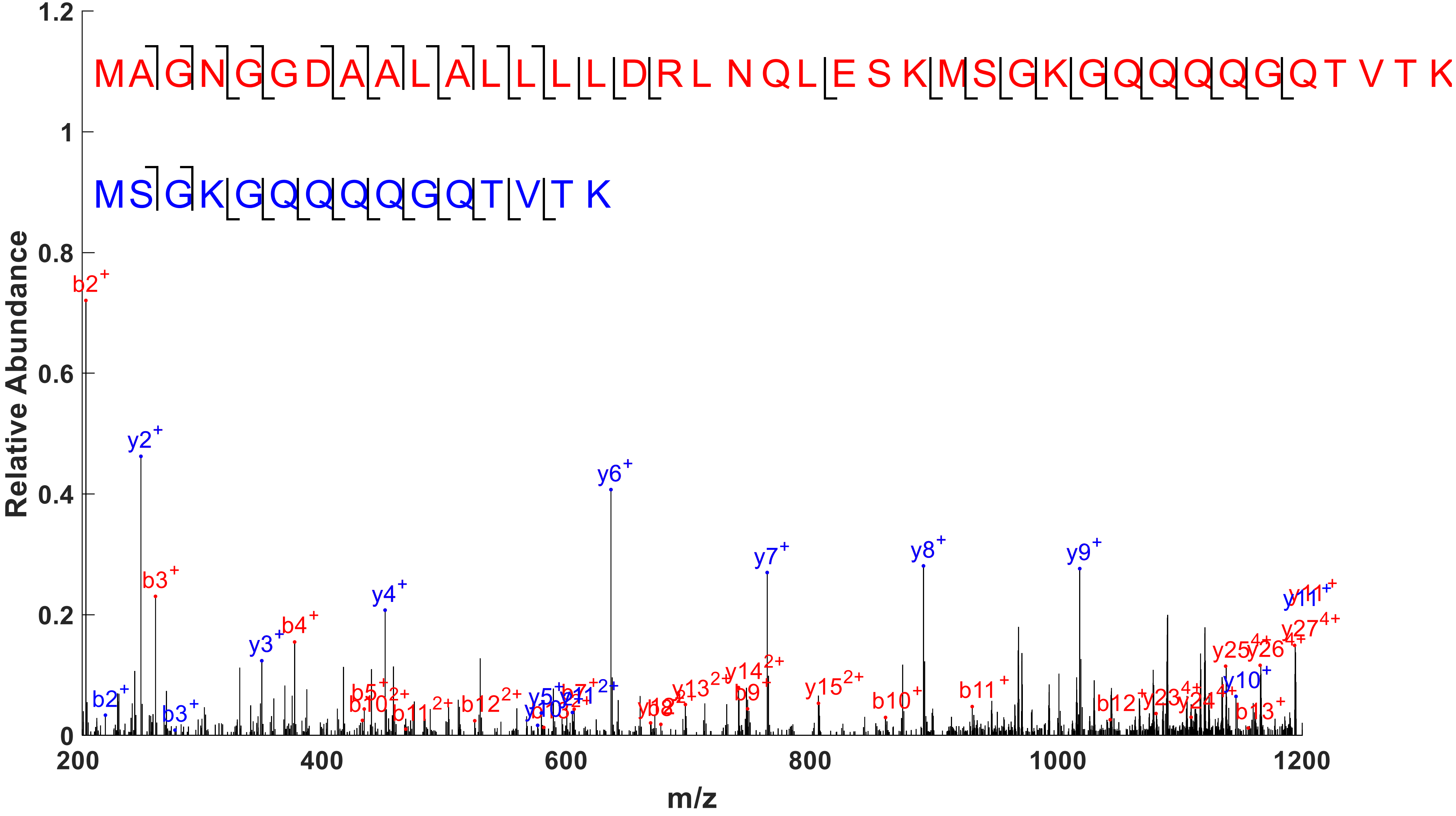

Protein N dimer cross link (4) K233 – K237.

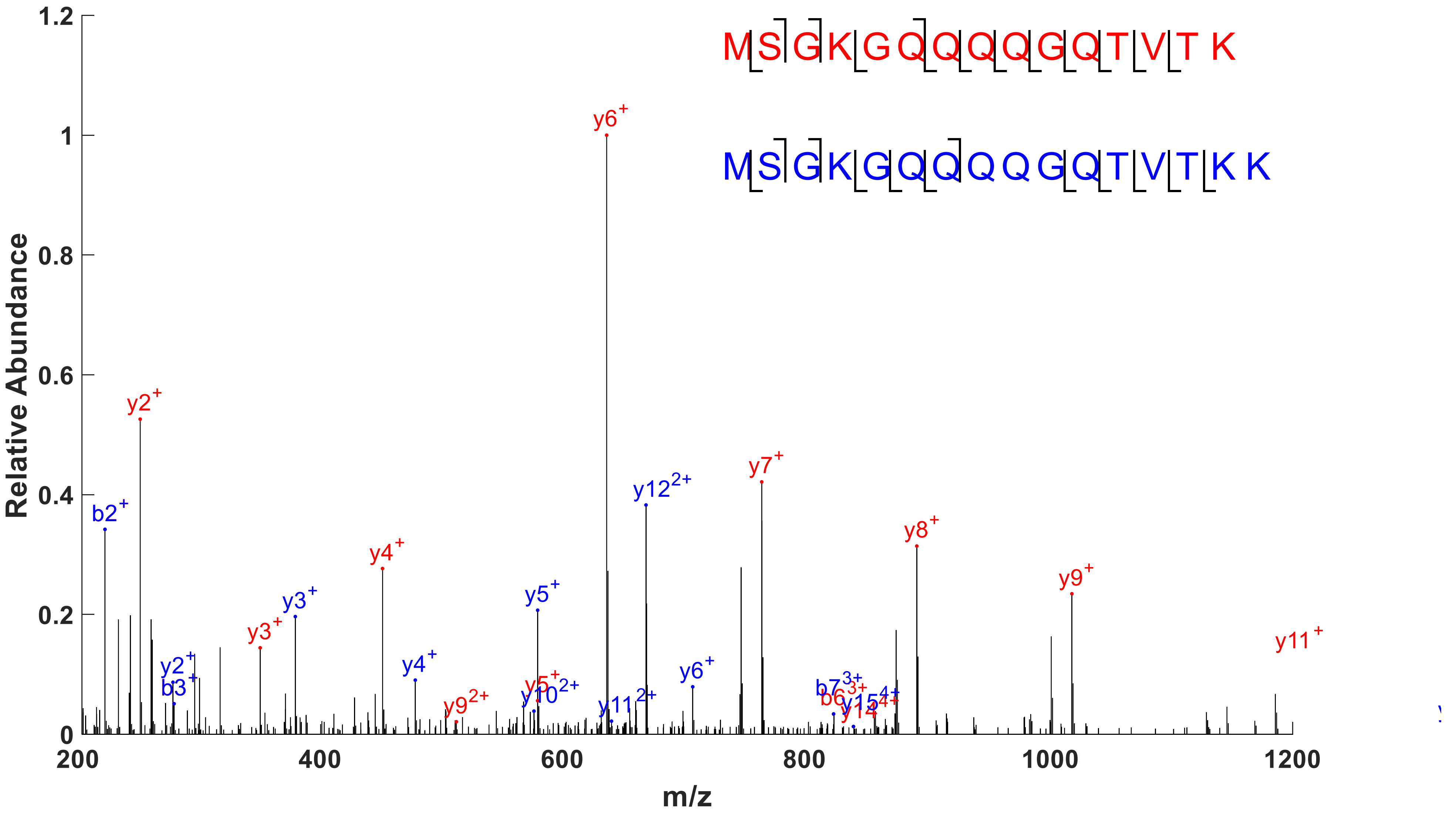

Protein N dimer cross link (5) K237 – K237.

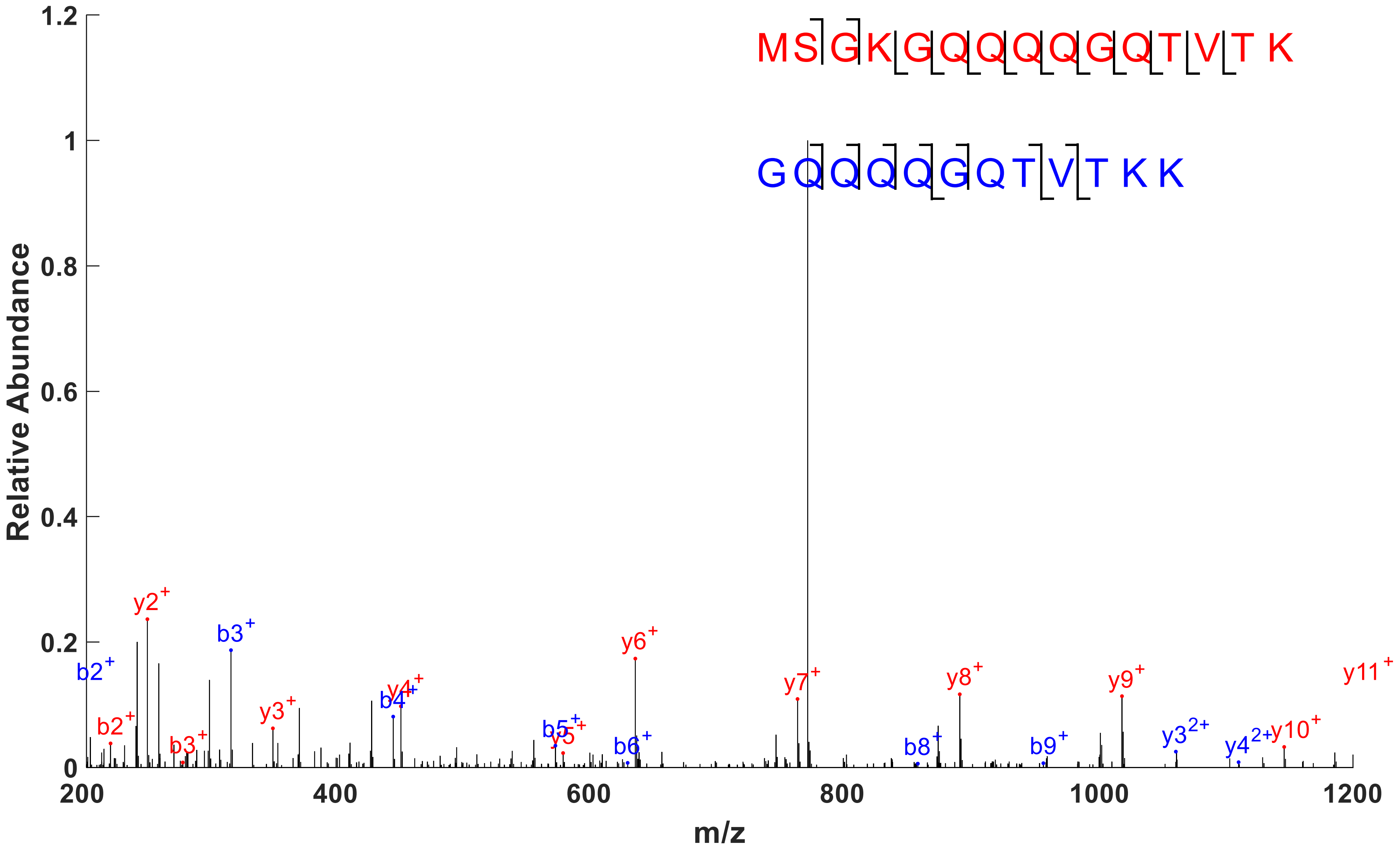

Protein N dimer cross link (6) K237 – K248.

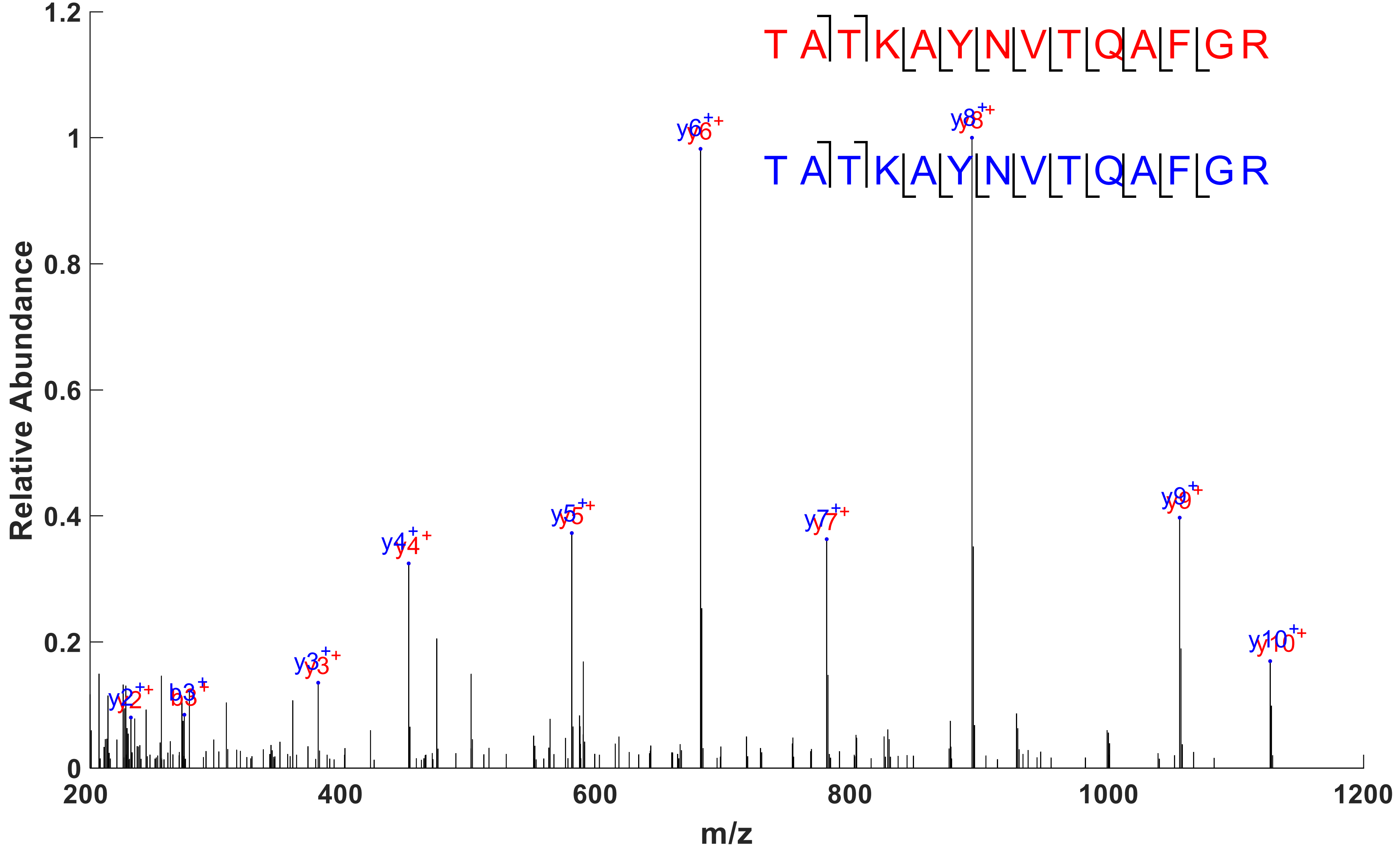

Protein N dimer cross link (7) K266 – K266.

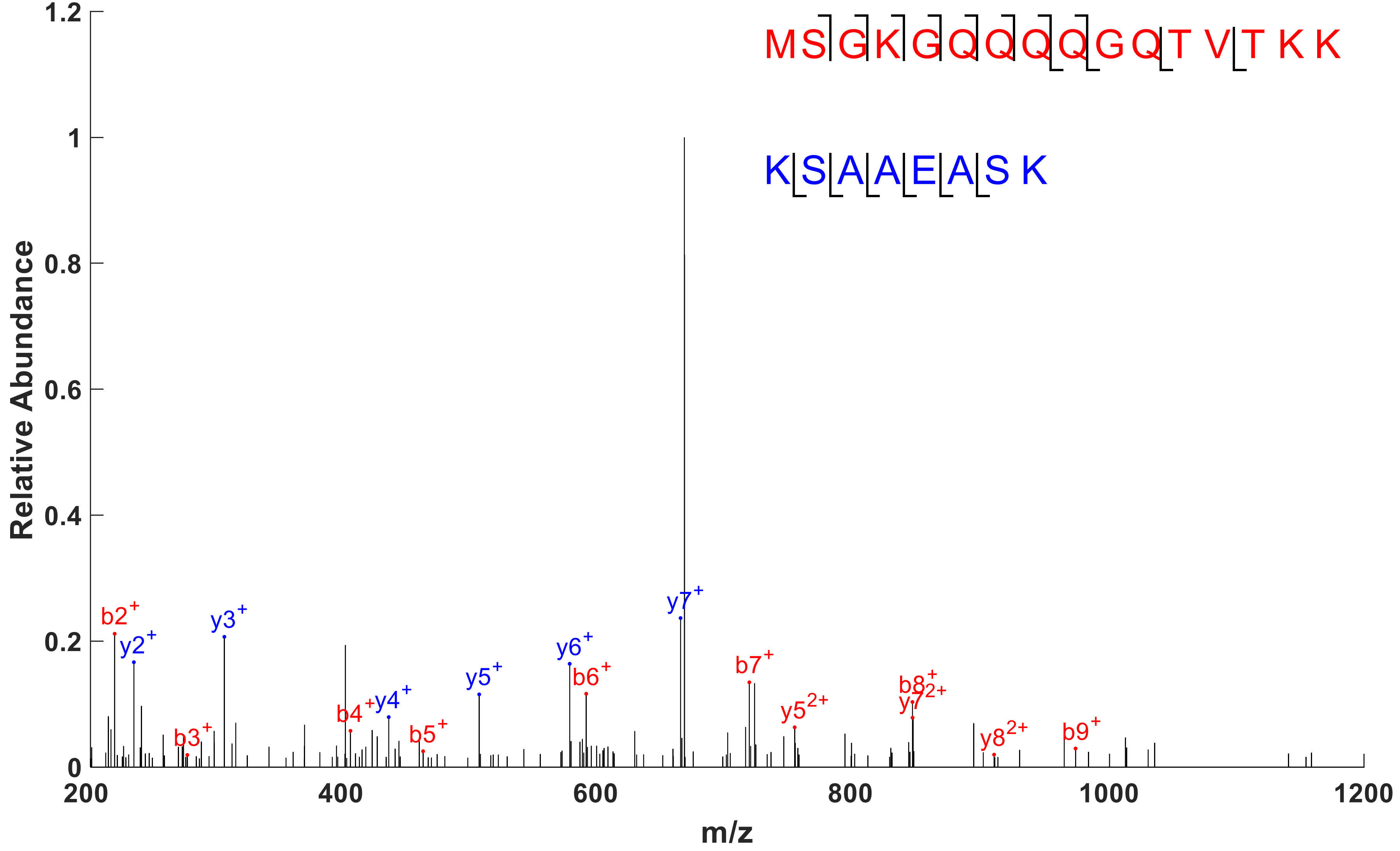

Protein N dimer cross link (8) K248 – K249.

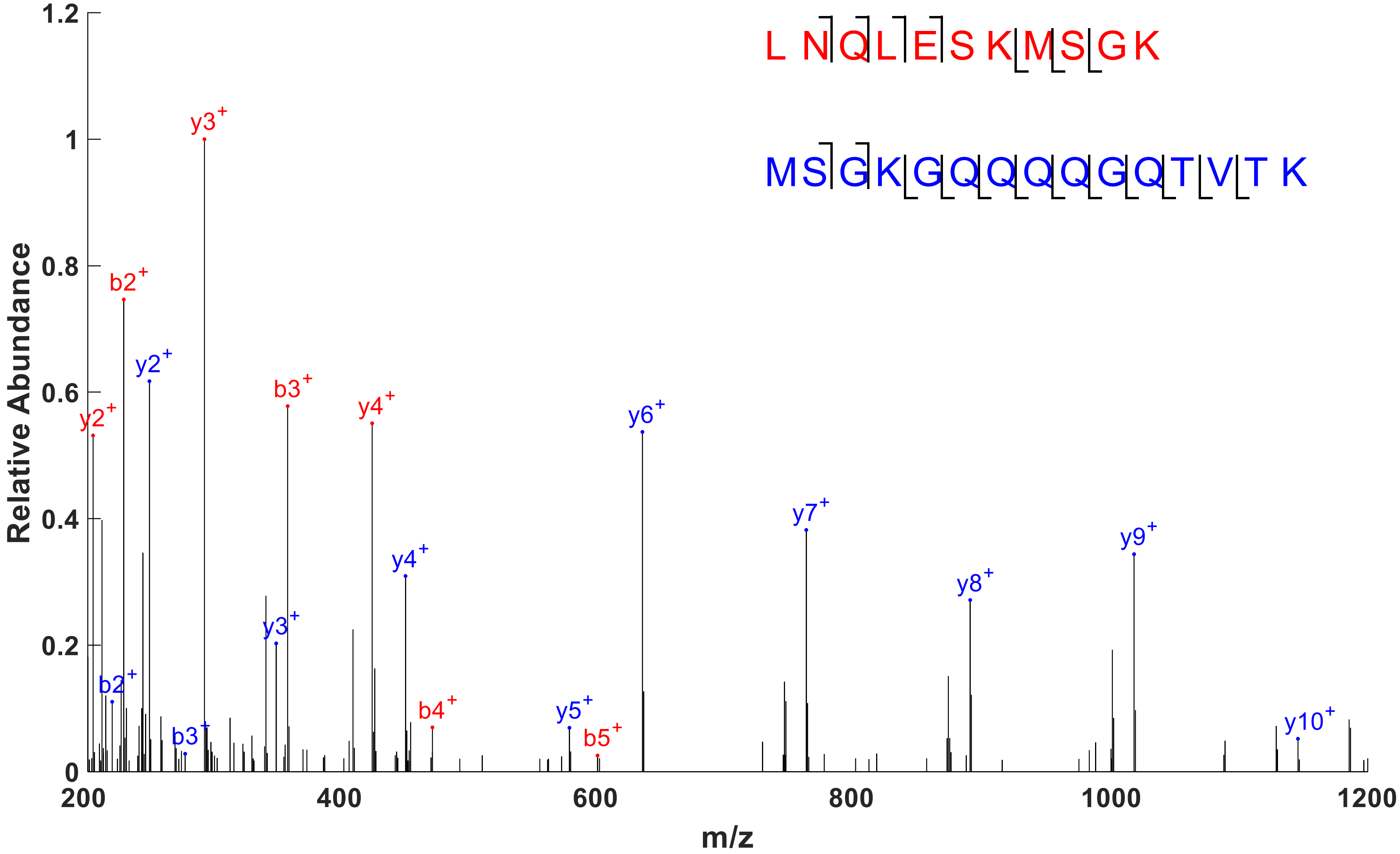

Protein N dimer cross link (9) K233 – K237.

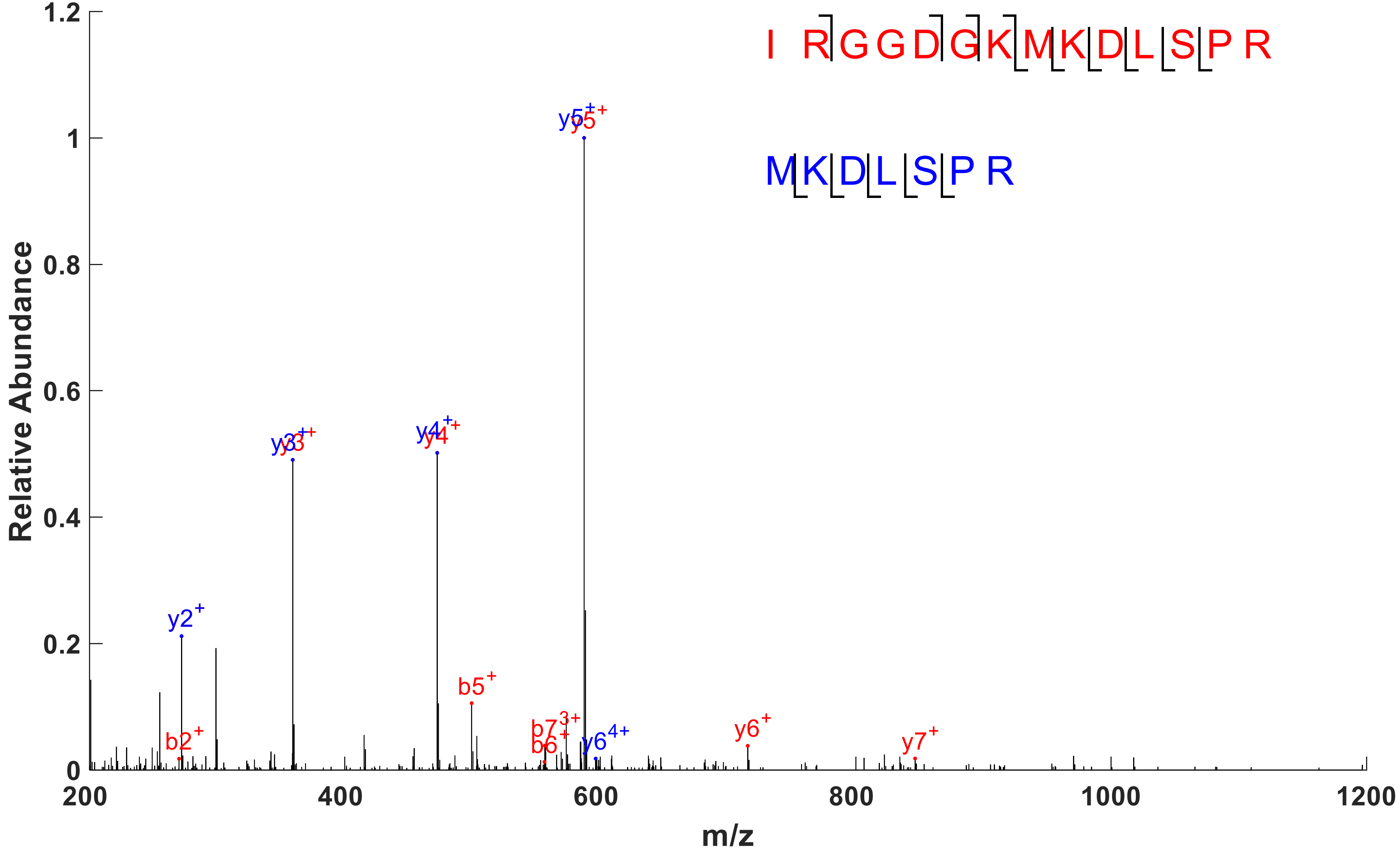

Protein N dimer cross link (10) K100 – K102.
